# Hap2–Ino80-facilitated transcription promotes de novo establishment of CENP-A chromatin

**DOI:** 10.1101/gad.332536.119

**Published:** 2020-02-01

**Authors:** Puneet P. Singh, Manu Shukla, Sharon A. White, Marcel Lafos, Pin Tong, Tatsiana Auchynnikava, Christos Spanos, Juri Rappsilber, Alison L. Pidoux, Robin C. Allshire

**Affiliations:** 1 Wellcome Centre for Cell Biology, School of Biological Sciences, The University of Edinburgh, Edinburgh EH9 3BF, United Kingdom;; 2 Bioanalytics, Institute of Biotechnology, Technische Universität Berlin, 13355 Berlin, Germany

**Keywords:** CENP-A, centromere, chromatin, chromosome segregation, epigenetic, fission yeast, histone, kinetochore, remodeling, transcription

## Abstract

Centromeres are maintained epigenetically by the presence of CENP-A, an evolutionarily conserved histone H3 variant, which directs kinetochore assembly and hence centromere function. To identify factors that promote assembly of CENP-A chromatin, we affinity-selected solubilized fission yeast CENP-A^Cnp1^ chromatin. All subunits of the Ino80 complex were enriched, including the auxiliary subunit Hap2. Chromatin association of Hap2 is Ies4-dependent. In addition to a role in maintenance of CENP-A^Cnp1^ chromatin integrity at endogenous centromeres, Hap2 is required for de novo assembly of CENP-A^Cnp1^ chromatin on naïve centromere DNA and promotes H3 turnover on centromere regions and other loci prone to CENP-A^Cnp1^ deposition. Prior to CENP-A^Cnp1^ chromatin assembly, Hap2 facilitates transcription from centromere DNA. These analyses suggest that Hap2–Ino80 destabilizes H3 nucleosomes on centromere DNA through transcription-coupled histone H3 turnover, driving the replacement of resident H3 nucleosomes with CENP-A^Cnp1^ nucleosomes. These inherent properties define centromere DNA by directing a program that mediates CENP-A^Cnp1^ assembly on appropriate sequences.

## Abstract (precis)

In this study, Singh et al. set out to define mechanisms for H3:CENP-A exchange at centromeric regions in *S. pombe.* Using a combination of biochemical, molecular, and genetic approaches, the authors show that Hap2–Ino80 destabilizes H3 nucleosomes on centromere DNA through transcription-coupled histone H3 turnover, driving the replacement of resident H3 nucleosomes with CENP-A nucleosomes.

The accurate delivery of all chromosomes to both resulting nuclei during mitotic cell division is required for eukaryotic cell viability and to prevent aneuploidy, a hallmark of cancer (Kops et al. 2005). The centromere region of chromosomes mediates their attachment to spindle microtubules for normal mitotic chromosome segregation (Fukagawa and Earnshaw 2014). In many organisms, centromeres are assembled on repetitive elements such as α-satellite repeats, minor satellite repeats, cen180/CentC/CentO repeats, and retroelements at human, mouse, plant, and *Drosophila* centromeres, respectively (Kipling et al. 1991; Grady et al. 1992; Cheng et al. 2002; Chang et al. 2019). Although such centromere repeats lack sequence similarity, in many cases their introduction as naked DNA into cells triggers de novo kinetochore assembly (Baum et al. 1994; Okada et al. 2007).

The underlying conserved feature at eukaryotic centromeres is the assembly of nucleosomes containing the histone H3 variant CENP-A (also generally known as cenH3, and specifically as CID in *Drosophila*, Cse4 in *Saccharomyces cerevisiae*, and Cnp1 in *Schizosaccharomyces pombe*) in place of canonical H3 to direct kinetochore assembly on such repetitive elements. Moreover, it is known that following deletion of an endogenous centromere, CENP-A incorporation and neocentromeres can arise at novel noncentromeric DNA locations (Ishii et al. 2008; Ketel et al. 2009; Shang et al. 2013). CENP-A chromatin has also been shown to be sufficient to trigger kinetochore assembly (Barnhart et al. 2011; Mendiburo et al. 2011; Hori et al. 2013; Chen et al. 2014). Thus, CENP-A deposition and not the primary sequence of centromere DNA determines the position of centromere formation. However, centromere DNA itself may harbor properties that favor CENP-A and kinetochore assembly (Baum et al. 1994; Fachinetti et al. 2015; Logsdon et al. 2019).

Three fission yeast species possess complex regional centromeres in which CENP-A^Cnp1^ and kinetochores are assembled over a nonrepetitive central domain of ∼10 kb in *S. pombe*, *S. octosporus*, and *S. cryophilus* (Tong et al. 2019). Although flanking gene order is preserved, the central domain sequence is not conserved among these species. Despite this lack of similarity, central domain DNA from *S. octosporus* and *S. cryophilus,* as well as *S. pombe*, can direct de novo CENP-A^Cnp1^ and kinetochore assembly in *S. pombe* (Folco et al. 2008; Catania et al. 2015; Tong et al. 2019). This suggests that these nonhomologous centromere DNAs possess innate features that program events that preferentially trigger the assembly of CENP-A^Cnp1^ in place of H3 nucleosomes.

During replication, parental CENP-A has been shown to distribute equally to nucleosomes on duplicated DNA of both sister centromeres, thus halving the amount of CENP-A at each centromere (Jansen et al. 2007; Schuh et al. 2007). Replenishment by deposition of newly synthesized CENP-A is temporally separated from DNA replication-coupled H3 chromatin assembly. The timing of new CENP-A deposition differs in various species; mitosis/late telophase/early G1 in human and *Drosophila* (Schuh et al. 2007; Erhardt et al. 2008; Mellone et al. 2011), S phase in *S. cerevisiae* (Pearson et al. 2004), and G2 in plants and *S. pombe* (Lermontova et al. 2006; Shukla et al. 2018).

Several studies have reported the association of RNA polymerase II (RNAPII) with and/or transcription from CENP-A-associated DNA so that noncoding transcription has become an apparent integral feature of centromeres (Duda et al. 2017). RNAPII has been detected at human artificial chromosome (HAC) (Bergmann et al. 2011), human metaphase (Chan et al. 2012), *Drosophila* (Bobkov et al. 2018), and *S. cerevisiae* (Ohkuni and Kitagawa 2011) centromeres, and also the central domains of fission yeast centromeres (Choi et al. 2011; Catania et al. 2015). Disruption of centromere transcription appears to hinder CENP-A loading and/or maintenance (Nakano et al. 2008; Bergmann et al. 2011; Chen et al. 2015; McNulty et al. 2017; Ling and Yuen 2019). Moreover, increased centromere transcription results in the rapid loss of CENP-A and centromere function (Hill and Bloom 1987; Bergmann et al. 2012). The central CENP-A^Cnp1^ domains from *S. pombe* centromeres contain numerous RNAPII transcriptional start sites and promoters (Choi et al. 2011; Catania et al. 2015). In addition, ectopically located central domain DNA, which lacks CENP-A^Cnp1^ chromatin, exhibits high rates of histone H3 turnover (Shukla et al. 2018). Such observations suggest that transcription-coupled chromatin remodeling events might drive the eviction of H3 and its replacement with CENP-A^Cnp1^. Consistent with this view, the accumulation of elongating RNAPII on ectopic centromere DNA during G2 coincides with the eviction of H3 and deposition of new CENP-A (Shukla et al. 2018). However, it remains to be determined which transcription-associated chromatin remodeling factors provoke the replacement of H3 with CENP-A on naïve centromere DNA.

Various ATP-dependent chromatin remodeling complexes provide access to the underlying DNA of chromatin-coated templates. Their activities enable transcription by disassembling nucleosomes, sliding nucleosomes, or replacing nucleosomal histone subunits with transcription-promoting variants (Clapier and Cairns 2009). The remodeling and spacing factor (RSF) interacts with CENP-A chromatin in mid-G1, its depletion reduces CENP-A levels at centromeres (Perpelescu et al. 2009), and tethering of RSF1 to centromere repeats promotes histone turnover/exchange resulting in both H3.3 and CENP-A deposition (Ohzeki et al. 2016). In *S. pombe*, Hrp1 (ortholog of chromo-helicase DNA-binding protein 1 [CHD1]) is enriched at centromeres and is required to maintain normal CENP-A^Cnp1^ levels at centromeres (Walfridsson et al. 2005; Choi et al. 2011). Loss of the histone chaperone facilitates chromatin transcription (FACT) results in promiscuous CENP-A^Cnp1^ assembly at noncentromeric locations A^Cnp1^ (Choi et al. 2012), suggesting that CENP-A^Cnp1^ may be titrated away from centromeres by loss of such factors. Moreover, inducible ectopic centromeres in *Drosophila* requires FACT-mediated RNAPII-dependent transcription of underlying DNA, indicating a necessity for transcription during CENP-A assembly (Chen et al. 2015).

Ino80 is a Snf2 family ATPase evolutionarily conserved from yeast to humans that participates in transcription, DNA replication, and DNA repair (Conaway and Conaway 2009). The Ino80 complex (Ino80C) can slide nucleosomes in an ATP-dependent manner (Chen et al. 2011) and can space multiple nucleosomes on longer DNA fragments (Udugama et al. 2011). Ino80C may also remove H2A.Z–H2B dimers from nucleosomes, replacing them with H2A–H2B dimers (Papamichos-Chronakis et al. 2011; Brahma et al. 2017). H2A.Z is enriched in +1 nucleosomes downstream from promoters of many active genes, and loss of Ino80 function affects transcription (Hogan et al. 2010; Marques et al. 2010; Xue et al. 2015). Individual Ino80C subunits make up three modules that associate with the main Ino80 ATPase subunit (Supplemental Table S1; Chen et al. 2011). *S. pombe* Ino80 has been shown to influence the maintenance of CENP-A^Cnp1^ chromatin at centromeres (Choi et al. 2017). However, it is not known whether Ino80C influences centromere DNA transcription or the establishment of CENP-A chromatin on naïve centromere DNA and, thus, centromere identity.

Here we used affinity selection of CENP-A^Cnp1^ chromatin and mass spectrometry to identify proteins enriched in CENP-A^Cnp1^ chromatin that may promote CENP-A^Cnp1^ assembly. We identify Hap2 (SPCC16C4.20) as an auxiliary subunit of Ino80C that is required for the conversion of H3 chromatin to CENP-A^Cnp1^ chromatin on naïve centromere DNA. We show that loss of Hap2 function reduces transcription and histone H3 turnover on centromere DNA. Our findings indicate that Hap2–Ino80 is required to promote transcription-associated chromatin remodelling events that drive H3 nucleosome eviction and the assembly of CENP-A^Cnp1^ nucleosomes in their place.

## Results

### Hap2 is an Ino80C subunit that is enriched in CENP-A^Cnp1^ chromatin

**Figure 1.**
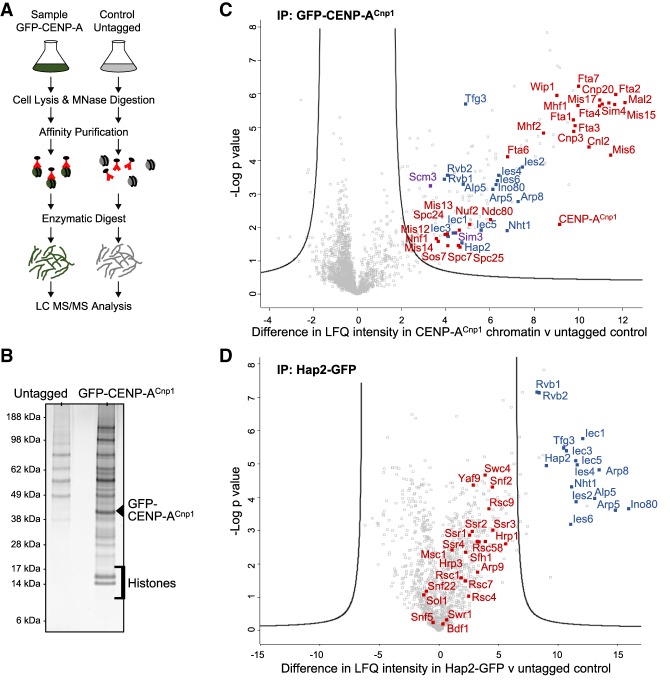
Hap2 is associated with CENP-A^Cnp1^ chromatin and it is a subunit of Ino80C. (*A*) Scheme for enrichment of CENP-A^Cnp1^ chromatin associated proteins and identification by mass spectrometry. (*B*) Silver-stained SDS-PAGE of proteins enriched in IPs from GFP-CENP-A^Cnp1^ or untagged control cells. (*C*) Volcano plot comparing label-free quantification (LFQ) intensity of proteins enriched in affinity selected GFP-CENP-A^Cnp1^ (anti-GFP) versus untagged control. Inner and outer kinetochore proteins detected (red). Ino80C subunits (blue). CENP-A^Cnp1^ chaperones Scm3 and Sim3 (purple). (*D*) Volcano plot comparing label-free quantification of proteins enriched in affinity selected Hap2-GFP (anti-GFP) versus untagged control. Ino80C subunits (blue). Swr1 complex subunits, SWI/SNF complex, RSC complex, and CHD family members (red).

To identify proteins involved in the assembly of CENP-A^Cnp1^ chromatin, GFP-tagged CENP-A^Cnp1^ chromatin was affinity-purified from micrococcal nuclease (MNase)-solubilized chromatin extracts (Fig. 1A). Quantitative PCR (qPCR) analysis of the resulting enriched native chromatin revealed significant enrichment of centromeric DNA from the single-copy central CENP-A^Cnp1^ domain of cen2 (cc2) compared with the 18 copies of flanking outer repeat (*dg*) sequences (Supplemental Fig. S1A). In addition, SDS-PAGE analysis showed core histones to be prevalent in this affinity-selected GFP-CENP-A^Cnp1^ material (Fig. 1B; Supplemental Fig. S1B). Label-free quantitative mass spectrometry detected strong enrichment of all known subunits of both the inner and outer kinetochore complexes (Fig. 1C; Supplemental Tables S2, S3). As our procedure also showed association of the chaperones Scm3 (HJURP) and Sim3 (NASP) that mediate CENP-A^Cnp1^ deposition we reasoned that other enriched, but non-centromere-specific, proteins might be involved in the incorporation of CENP-A^Cnp1^ into centromeric chromatin.

It was notable that all subunits of the Ino80 chromatin remodeling complex (Ino80C) were enriched in our affinity-selected GFP-CENP-A^Cnp1^ preparations (Fig. 1C). Also enriched in these samples was the low-molecular-weight α-helical Hap2 protein. Hap2 has previously been reported to associate with Ino80C but no characterization has been performed (Hogan et al. 2010). To confirm association of Hap2 with Ino80C, Hap2 was C-terminally tagged with GFP at its endogenous chromosomal locus (Hap2-GFP) (Supplemental Fig. S2A). Affinity selection of Hap2-GFP followed by proteomic analysis revealed that all subunits of Ino80C, but not subunits of other remodelling complexes, are enriched with Hap2-GFP (Fig. 1D; Supplemental Table S4). IP-western analysis confirmed that Hap2-GFP associates with immunoprecipitated HA-tagged Ino80 (Ino80-HA) and vice-versa, independently of DNA/chromatin association (Supplemental Fig. S1C,D). Furthermore, affinity selection of Ino80-HA enriched all known Ino80C subunits along with Hap2 (Supplemental Fig. S1E; Supplemental Table S5). We conclude that Hap2 is a noncanonical subunit of the fission yeast Ino80 complex and that all Ino80C subunits are enriched in CENP-A^Cnp1^ chromatin.

### Hap2 association with central domain DNA assembled in CENP-A^Cnp1^ chromatin or H3 chromatin is Ies4-dependent

**Figure 2.**
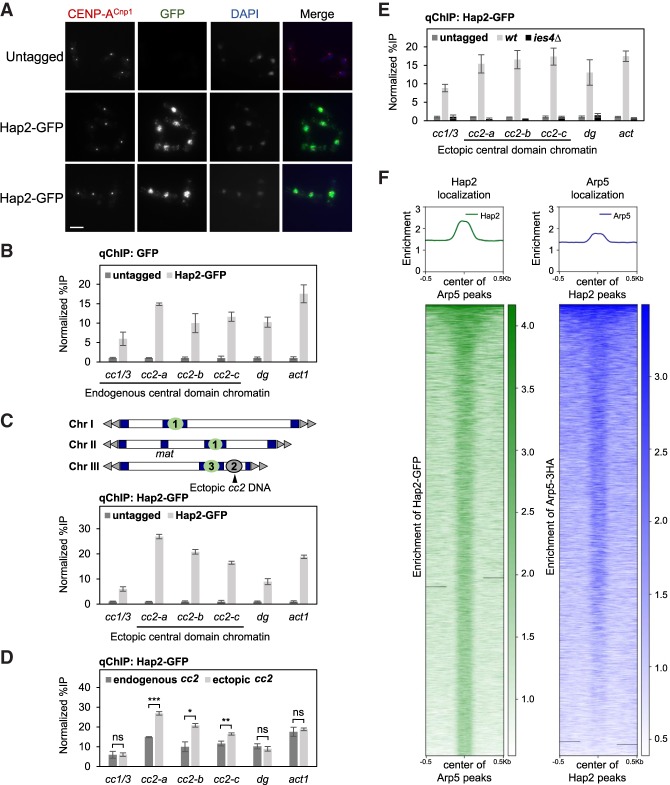
Hap2 associates with endogenous and ectopic central core chromatin and Ies4 dependent, Hap2 and Arp5 chromosomal distributions are coincident. (*A*) Immunolocalization of Hap2-GFP in cells. Representative images of wild-type and Hap2-GFP cells stained with anti-CENP-A^Cnp1^ (red), anti-GFP (green), and DAPI (blue). Scale bar, 5 µm. (*B*) qChIP for Hap2-GFP at four locations within endogenous centromeres (*cc1/3*, *cc2-a*, *cc2-b*, and *cc2-c*), outer repeat heterochromatin (*dg*) and noncentromere locus (*act1*^+^). Error bars indicate mean ± SD (*n* = 3). (*C*, *top*) Diagram indicating strain with endogenous *cen2-cc2* replaced with *cen1* central domain DNA (*cc1*) at the noncentromeric ura4 locus (*ura4:cc2*) on Chr III. (*Bottom*) qChIP for Hap2-GFP at three locations within ectopic central domain H3 chromatin (*cc2-a*, *cc2-b*, and *cc2-c*), endogenous centromeres (*cc1/3*), outer repeat heterochromatin (*dg*), and noncentromere locus (*act1*^+^). Error bars indicate mean ± SD (*n* = 3). (*D*) Comparison of Hap2-GFP association with central domain sequence assembled in CENP-A^Cnp1^ chromatin or H3 chromatin (data from *B* and *C*). Error bars indicate mean ± SD (*n* = 3). Significance of the differences observed between cells containing endogenous centromeres and ectopic central domain DNA was evaluated using Student's *t*-test. (*) *P* < 0.05; (**) *P* < 0.005; (***) *P* < 0.0005; (n.s.) not significant. (*E*) qChIP for Hap2-GFP at three locations within ectopic central domain H3 chromatin (*cc2-a*, *cc2-b*, and *cc2-c*), endogenous centromeres (*cc1/3*), outer repeat heterochromatin (*dg*) and noncentromere locus (*act1*^+^) in untagged, wild-type, and *ies4*Δ cells. (*F*) Genome-wide coincidence of Hap2-GFP and Arp5-3HA peaks (∼60%). Gradient shows enrichment of Hap2 (green) and Arp5 (blue).

Microscopic analysis showed that Hap2-GFP localizes to the nucleus (Fig. 2A). Quantitative chromatin immunoprecipitation (qChIP) assays revealed that Hap2-GFP associates with most chromatin regions analyzed including the central CENP-A^Cnp1^ domain of centromeres (*cc2*), the flanking pericentromeric outer repeats (*dg*) and on the highly expressed *act1*^+^ gene (Fig. 2B). When 8.5 kb of cen2 central domain DNA (*cen2-cc2*) is inserted at the *ura4* locus on a chromosome arm (*ura4:cc2*), it is assembled in H3 instead of CENP-A^Cnp1^ chromatin (Choi et al. 2012; Shukla et al. 2018). Replacement of 6.5 kb of endogenous *cen2-cc2* DNA with 5.5 kb of *cen1* central domain DNA (*cc2Δ::cc1*) allows analysis across the resulting unique ectopic copy of cen2 central domain DNA (*ura4:cc2*) in the absence CENP-A^Cnp1^ and kinetochore proteins (Fig. 2C, top). qChIP analysis revealed that Hap2-GFP associates with this ectopic *cc2* central domain chromatin (Fig. 2C, bottom). A noticeably higher level of Hap2-GFP was consistently detected across ectopic *ura4:cc2* central domain DNA assembled in H3 chromatin relative to the same DNA sequence at the native *cen2* central domain assembled in CENP-A^Cnp1^ chromatin (Fig. 2D). Loss of Hap2 does not affect the association of Ino80-HA with these regions (Supplemental Fig. S2B), while Hap2 chromatin association is lost in the absence of Ies4 but remains unaffected in *iec1*Δ, *ies2*Δ and *arp5*Δ cells (Fig. 2E; Supplemental Fig. S2C,D). In budding yeast, different subunits of Ino80 have been shown to have broad interactions around NFRs and +1 nucleosomes (Yen et al. 2013). As Hap2 is a subunit of Ino80C, we examined genome-wide localization of Hap2 and Arp5. High coincidence was observed for Hap2 and Arp5 peaks (Fig. 2F). We conclude that the Ino80C subunit Hap2 is a nuclear protein that associates with noncentromeric loci and is preferentially recruited to centromeric central domain chromatin when assembled in H3 rather than CENP-A^Cnp1^ chromatin. Hap2 chromatin association is dependent on the Ino80C subunit Ies4, and Hap2 colocalizes genome-wide with the Arp5 canonical Ino80C subunit.

### Hap2 is required to maintain CENP-A^Cnp1^ chromatin across endogenous centromeres

**Figure 3.**
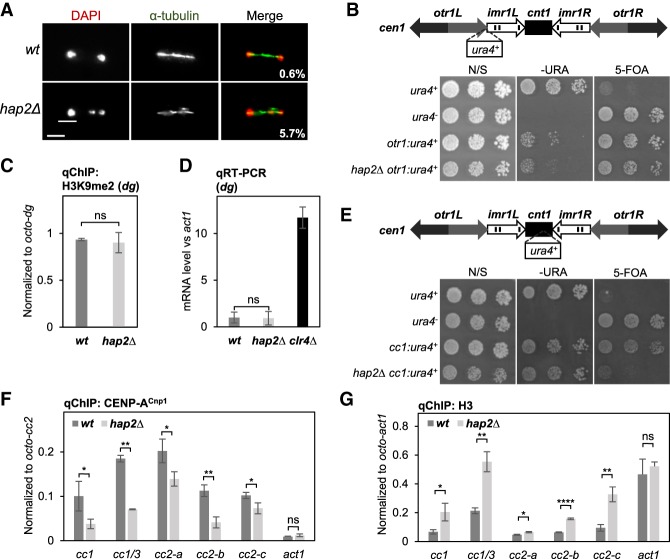
Hap2 is required to maintain CENP-A^Cnp1^ chromatin but not the adjacent pericentric heterochromatin. (*A*) Frequency of lagging chromosomes in anaphase cells. Representative images of wild-type and *hap2*Δ cells stained with DAPI (red) and antitubulin (green). Percentage of late anaphase cells (wild type: *n* = 487 and *hap2*Δ: *n* = 492) displaying lagging chromosomes is indicated. Scale bar, 5 µm. (*B*, *top*) Diagram indicating position *otr1:ura4*^+^ marker gene inserted in outer repeat heterochromatin of *cen1*. (*Bottom*) Growth assay for central domain silencing at *otr1:ura4*^+^. Fivefold serial dilution of cell cultures were spotted on nonselective (N/S), selective (−URA), or counter-selective (FOA) plates. (*C*) qChIP for H3K9me2 at outer repeat heterochromatin (*dg*). Error bars indicate mean ± SD (*n* = 3). (*D*) qRT-PCR of pericentric *dg* repeats performed on total RNA extracted from indicated strains. Transcript levels are shown relative to *act1*^+^ and normalized to wild type (*n* = 3). (*E*, *top*) Diagram indicating position *cc1:ura4*^+^ marker gene within the central CENP-A^Cnp1^ domain *cen1 (cnt1)* relative to the outer repeat (*otr*) and innermost repeats (*imr*). (*Bottom*) Growth assay for central domain silencing at *cen1:ura4*^+^ as in *B*. (*F*,*G*) qChIP for CENP-A^Cnp1^ (*F*) and histone H3 (*G*) at five locations within endogenous centromeres (*cc1*, cc1/3, *cc2-a*, *cc2-b*, and *cc2-c*) and noncentromere locus (*act1*^+^). Error bars indicate mean ± SD (*n* = 3). Significance of the differences observed between wild-type and *hap2*Δ was evaluated using Student's *t*-test in *C*, *D*, *F*, and *G*. (*) *P* < 0.05; (**) *P* < 0.005; (****) *P* < 0.00005; (n.s.) not significant.

Cells lacking Hap2 exhibit an elevated frequency of lagging chromosomes during mitosis, indicating that loss of Hap2 may affect centromere function (Fig. 3A). Defective centromere function can result from reduced pericentromeric heterochromatin formation on *dg*/*dh* outer repeats or CENP-A^Cnp1^ chromatin/kinetochore assembly and these can be sensitively detected by the use of silent *ura4*^+^ reporter genes inserted within outer repeat heterochromatin or central CENP-A^Cnp1^ domain chromatin (Allshire et al. 1994, 1995; Partridge et al. 2000). No alleviation of heterochromatin-mediated silencing at *otr1:ura4*^+^ was detected in *hap2*Δ cells relative to wild type as indicated by similar poor growth on selective plates lacking uracil (−URA) and good growth on counter-selective 5-FOA plates (Fig. 3B; Supplemental Fig. S3A). Consistent with this observation, no significant change was detected in the levels of the heterochromatin H3K9me2 mark or *dg* transcripts produced by the underlying outer repeats in *hap2*Δ relative wild-type cells (Fig. 3C,D).

Central CENP-A^Cnp1^ domain chromatin is also transcriptionally repressive (Allshire et al. 1994, 1995). Defects in CENP-A^Cnp1^ chromatin assembly alleviates this transcriptional silencing (Partridge et al. 2000; Pidoux et al. 2003). To test whether loss of Hap2 affects CENP-A^Cnp1^-mediated silencing we examined silencing of *ura4*^+^ embedded in central CENP-A^Cnp1^ chromatin at *cen1* (*cc1:ura4*^+^). Reduced silencing in *hap2*Δ cells, indicated by reduced growth on counter-selective FOA plates, suggested a defect in CENP-A^Cnp1^ chromatin integrity (Fig. 3E; Supplemental Fig. S3B). qChIP analysis detected significantly lower levels of CENP-A^Cnp1^ and a reciprocal increase in H3 levels across the central domain of centromeres in *hap2*Δ relative to wild-type cells (Fig. 3F,G), consistent with the silencing defect. Importantly, loss of Hap2 does not affect the total cellular levels of GFP-CENP-A^Cnp1^ or the expression of genes encoding proteins that are known to regulate CENP-A^Cnp1^ loading at centromeres (Supplemental Fig. S3C,D). We conclude that loss of Hap2 specifically affects silencing through loss of CENP-A^Cnp1^ and gain of H3 within the central domain of centromeres, thus Hap2 is required to maintain CENP-A^Cnp1^ chromatin integrity at endogenous centromeres.

### Hap2 is required for the de novo establishment of CENP-A^Cnp1^ chromatin

**Figure 4.**
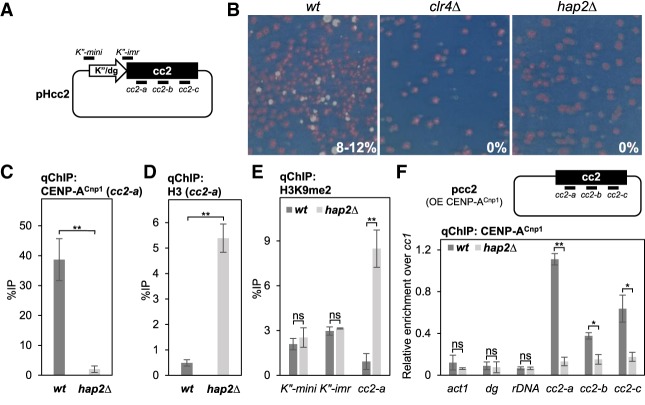
Hap2 is required for the de novo establishment of CENP-A^Cnp1^ chromatin. (*A*) Schematic representation of pHcc2 minichromosome K″/dg repeat adjacent to central domain 2 DNA. Position of the primer pair at the edge of K″/dg repeat and within central domain 2 DNA are indicated. (*B*) Transformants containing pHcc2 minichromosome plasmids were replica-plated to low adenine nonselective plate. Representative plate showing colony color and centromere establishment frequency in wild type (*n* = 515), *hap2*Δ (*n* = 390), and strains lacking heterochromatin *clr4*Δ (*n* = 870). (*C*,*D*) qChIP for CENP-A^Cnp1^ and histone H3 at plasmid-borne central domain 2 DNA (*cc2-a*) on pHcc2 minichromosome. Error bars indicate mean ± SD (*n* = 3). (*E*) qChIP for H3K9me2 at plasmid-borne K″/dg repeat (*K″-mini* and *K″-imr*) and plasmid-borne central domain 2 DNA (*cc2-a*) on pHcc2 minichromosome. Error bars indicate mean ± SD (*n* = 3). (*F*, *top*) Schematic representation of pcc2 minichromosome without K″/dg repeat transformed in cells overexpressing CENP-A^Cnp1^. (OE) Overexpression. (*Bottom*) qChIP for CENP-A^Cnp1^ at noncentromere locus (*act1*^+^), outer repeat heterochromatin (*dg*) and ribosomal DNA (*rDNA*), and three locations within plasmid-borne central core 2 DNA (*cc2-a*, *cc2-b*, and *cc2-c*) on pcc2 minichromosome relative to CENP-A^Cnp1^ levels at endogenous centromeres (*cc1/3*). Error bars indicate mean ± SD (*n* = 3). Significance of the differences observed between wild type and *hap2*Δ was evaluated using Student's *t*-test in *C*–*F*. (*) *P* < 0.05; (**) *P* < 0.005; (n.s.) not significant.

Many factors are known to assist CENP-A maintenance but little is known about the factors required for the de novo establishment of CENP-A chromatin. De novo establishment of functional centromeres can occur following the introduction of naked centromere DNA into cells (Catania et al. 2015). We first examined whether Hap2 and other subunits of Ino80C are required for the de novo establishment of centromeres on the pHcc2 minichromosome (Fig. 4A). *hap2*Δ cells exhibited a complete failure to establish functional centromeres, similar to *clr4*Δ cells that lack heterochromatin, while *ies2*Δ displayed increased establishment, *iec1*Δ and *ies4*Δ were less competent in establishing functional centromeres on pHcc2 (Fig. 4B; Supplemental Fig. S4A). In *S. pombe*, de novo CENP-A^Cnp1^ chromatin establishment on circular plasmid-based minichromosomes requires a block of heterochromatin in close proximity to central domain DNA (Folco et al. 2008; Kagansky et al. 2009). Loss of centromere establishment could result from a failure to establish CENP-A^Cnp1^ chromatin, and/or adjacent heterochromatin, on the minichromosome. To distinguish between these possibilities, CENP-A^Cnp1^ levels on the plasmid-borne central domain cc2 DNA were analysed. All strains used have 6 kb of *cen2* central domain DNA replaced with 5.5 kb of *cen1* central domain DNA at endogenous centromeres (*cc2Δ::cc1*) so that the plasmid-borne cc2 is the only copy of this element. qChIP revealed that CENP-A^Cnp1^ was not assembled over cc2 carried by pHcc2 in *hap2*Δ cells (Fig. 4C). Reciprocally, a high level of H3 was detected across cc2 of pHcc2 in the absence of CENP-A^Cnp1^ assembly in *hap2*Δ cells (Fig. 4D). qChIP for H3K9me2 demonstrated that loss of Hap2 did not affect the establishment of heterochromatin on the plasmid-borne K″/dg repeat (Fig. 4E). Interestingly, high levels of H3K9me2 were detected within the central domain of the pHcc2 minichromosome in *hap2*Δ but not wild-type cells (Fig. 4E). This suggests that in the absence of CENP-A^Cnp1^ chromatin establishment in *hap2*Δ cells heterochromatin may spread from the outer K″/dg repeat into the plasmid-borne central cc2 domain. To test whether Hap2 is required for the de novo establishment of CENP-A^Cnp1^ chromatin independently from the requirement for adjacent heterochromatin, the plasmid pcc2 which carries 8.5 kb of *cen2* central domain DNA, but no heterochromatin forming outer repeat sequences, was transformed into cells expressing additional GFP-CENP-A^Cnp1^, which allows CENP-A^Cnp1^ chromatin assembly (Fig. 4F, top; Catania et al. 2015). In contrast to wild type, *hap2*Δ cells did not assemble high levels of CENP-A^Cnp1^ over the central domain of the pcc2 minichromosome (Fig. 4F, bottom). This effect was not due to altered CENP-A^Cnp1^ protein levels in *hap2*Δ compared with wild-type cells (Supplemental Fig. S4B). We conclude that Hap2 is critical for the de novo establishment of CENP-A^Cnp1^ chromatin on naïve central domain centromere DNA.

### Hap2 promotes histone turnover in genomic regions prone to CENP-A^Cnp1^ assembly

**Figure 5.**
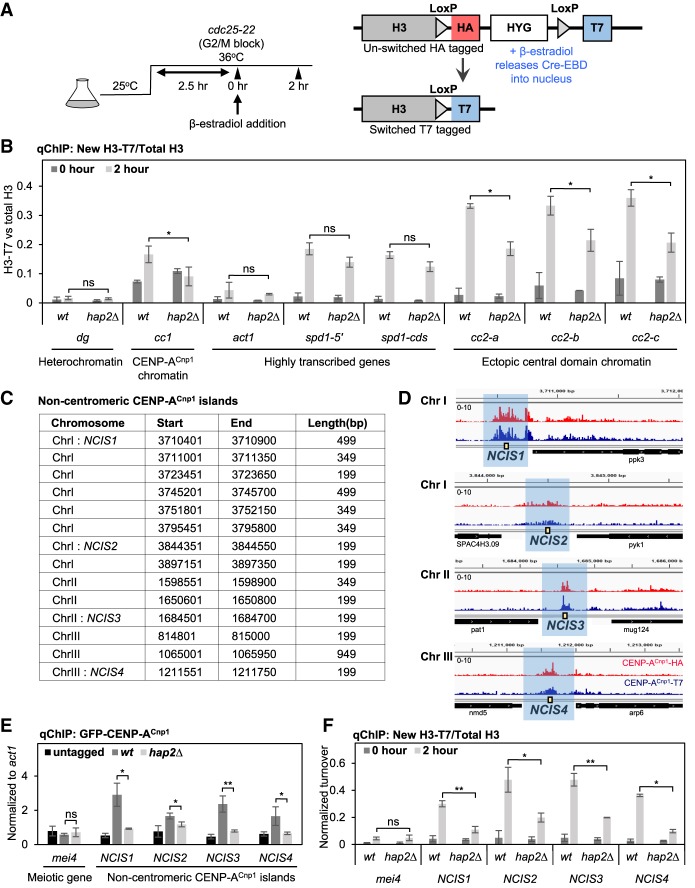
Hap2 is required for high histone H3 turnover on central domain centromere DNA and islands of noncentromeric CENP-A incorporation. (*A*) Experimental setup to assess replication-independent H3 turnover. The c*dc25-22* temperature sensitive mutation was used to block wild-type and *hap2*Δ cells in G2, the HA tag on H3 was swapped for the T7 tag by β-estradiol induced Cre/loxP mediated recombination. Samples were collected at 0 and 2 hr and analyzed by qChIP. (*B*) qChIP analysis of new H3.2-T7 incorporated in *cdc25-22*/G2-arrested cells at pericentromeric outer repeat heterochromatin (*dg*), the central CENP-A^Cnp1^ domain of cen1 (*cc1*), highly transcribed genes (*act1*^+^ and *spd1*^+^) and three locations within ectopically located *ura4:cc2* lacking CENP-A^Cnp1^ (*cc2-a*, *cc2-b*, and *cc2-c*). (*Y*-axis) H3.2-T7%IP values normalized to the respective total H3%IP values represent normalized turnover for each sample. Error bars indicate mean ± SD (*n* = 3). (*C*) ChIP-nexus analysis of HA and T7 tagged CENP-A^Cnp1^ reveals consistent noncentromeric CENP-A^Cnp1^ islands (NCIS) of incorporation above background at 14 locations in the genome. (*D*) Four CENP-A^Cnp1^ islands are shown: *NCIS1*, *NCIS2*, *NCIS3,* and *NCIS4*. (*E*) qChIP for GFP-CENP-A^Cnp1^ at noncentromeric CENP-A^Cnp1^ islands using indicated primer pairs (yellow boxes in *D*) and meiotic-specific gene (*mei4*^+^). Error bars indicate mean ± SD (*n* = 3). (*F*) qChIP analysis of new H3.2-T7 incorporation in *cdc25-22*/G2-arrested cells at *NCIS1*, *NCIS2*, *NCIS3*, and *NCIS4*. Error bars indicate mean ± SD (*n* = 3). Error bars indicate mean ± SD (*n* = 3). Significance of the differences observed between wild type and *hap2*Δ was evaluated using Student's *t*-test in *B*, *E*, and *F*. (*) *P* < 0.05; (**) *P* < 0.005; (n.s.) not significant.

Central domain DNA inserted at a noncentromeric location on the arm of a chromosome, such as the *ura4* locus on chromosome 3 (*ura4:cc2*), remains assembled in H3 nucleosomes and exhibits a high rate of histone H3 turnover (Shukla et al. 2018). The inherent instability of H3 nucleosomes assembled on this ectopic *ura4:cc2* centromere DNA has been proposed to aid the incorporation of CENP-A^Cnp1^ when available (Shukla et al. 2018). In *S. cerevisiae*, Ino80 associates with promoter-associated nucleosome-depleted regions and transcription start sites (TSS) where it mediates the turnover of +1 nucleosomes (Yen et al. 2013). The requirement for Hap2 in de novo CENP-A^Cnp1^ assembly on central domain DNA may result from defective H3 nucleosome turnover on these centromeric sequences when assembled in H3 chromatin alone. We therefore used recombination-induced tag exchange (RITE) (Verzijlbergen et al. 2010; Shukla et al. 2018) to measure replication-independent H3 turnover in G2-arrested wild-type and *hap2*Δ cells on ectopic *ura4:cc2,* heterochromatic repeats, and highly transcribed genes. This H3.2-HA→T7 tag swap was induced in *cdc25-22*/G2-arrested cells and the incorporation of new histone H3.2-T7 was monitored (Fig. 5A). Importantly, the H3.2-HA → T7 tag swap efficiency was unaffected by *hap2*Δ relative to wild-type cells (Supplemental Fig. S5A). Compared with wild-type cells, a significant decrease in the level of H3 turnover was evident on ectopic *ura4:cc2* centromere DNA in *hap2*Δ cells (Fig. 5B). Similarly, H3 turnover within endogenous *cen1-cc1* assembled in CENP-A^Cnp1^ chromatin was also reduced in *hap2*Δ cells. In contrast, H3 turnover remained unchanged within heterochromatic outer repeats (*dg*) and over highly transcribed genes (*act1*^+^ and *spd1*^+^). Ino80 may mediate nucleosome turnover through eviction of H2A.Z (Papamichos-Chronakis et al. 2011). However, the levels of H2A.Z^Pht1^ associated with endogenous *cc1* and ectopic *ura4:cc2* were unaffected by loss of Hap2 (Supplemental Fig. S5B). We conclude that Hap2 is required to ensure H3 nucleosome instability on centromeric sequences by mediating a high frequency of H3 turnover, which may consequently allow the incorporation of CENP-A^Cnp1^ in place of H3.

Overexpression of CENP-A^Cnp1^ results in low levels of promiscuous CENP-A^Cnp1^ incorporation at noncentromeric locations (Choi et al. 2012; Castillo et al. 2013). Fission yeast CENP-A^Cnp1^ expression is known to increase prior to that of canonical histones in advance of replication. Consequently, even without overexpression, in early S phase this natural excess of CENP-A^Cnp1^ results in low levels of newly synthesized CENP-A^Cnp1^ being incorporated across many gene bodies (Shukla et al. 2018). ChIP-Nexus (a modified exo-ChIP-seq protocol) (He et al. 2015) analysis allowed detection of noncentromeric genomic regions where islands of CENP-A^Cnp1^ are retained at low levels (Fig. 5C,D). Hap2-GFP was found to associate with these noncentromeric CENP-A^Cnp1^ islands (NCIS), while loss of Hap2 significantly decreased CENP-A^Cnp1^ incorporation within these islands (Fig. 5E). Consistent with previous results, Hap2 association with NCIS requires Ies4, but not Iec1, Ies2, or Arp5 (Supplemental Fig. S5C,D). Interestingly, qChIP also revealed reduced histone H3 turnover within these noncentromeric CENP-A^Cnp1^ islands in *hap2*Δ cells, whereas histone H3 turnover remained unchanged within the heterochromatic outer repeats, the *act1*^+^ gene, or the repressed meiosis-specific *mei4*^+^ gene (Fig. 5F). We conclude that Hap2 also promotes a high rate of H3 turnover at several noncentromeric NCIS loci prone to low-level CENP-A^Cnp1^ incorporation. This observation reinforces the involvement of Hap2–Ino80C in mediating histone H3 turnover and the exchange of histone H3 for CENP-A^Cnp1^-containing nucleosomes.

### Hap2 facilitates transcription of central domain chromatin

**Figure 6.**
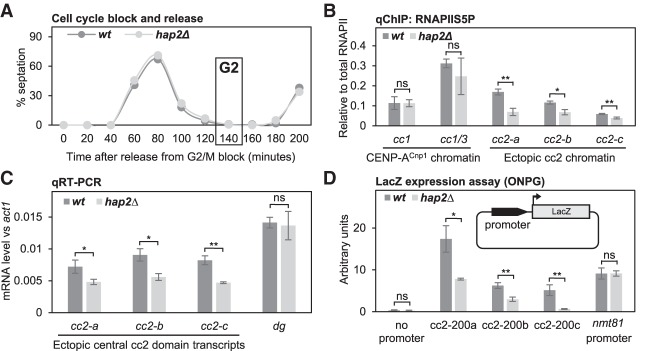
Transcription from centromeric DNA is reduced in the absence of Hap2. (*A*) *cdc25-22* synchronized cell populations were used to assess levels of RNAPIIS5P and central core transcripts during G2 in *B* and *C*. The septation index for wild type and *hap2*Δ were measured and cells were collected in G2 (T-140). (*B*) qChIP for initiating RNAPIIS5P at two locations within endogenous centromeres (*cc1* and *cc1/3*) and three locations within ectopically located *ura4:cc2* lacking CENP-A^Cnp1^ (*cc2-a*, *cc2-b*, and *cc2-c*) in wild type and *hap2*Δ. Error bars indicate mean ± SD (*n* = 3). (*C*) qRT-PCR to quantify transcripts from three locations within ectopically located *ura4:cc2* lacking CENP-A^Cnp1^ (*cc2-a*, *cc2-b*, and *cc2-c*) and from pericentromeric outer repeat (*dg*) on total RNA extracted from wild-type and *hap2*Δ cells. Transcript levels are shown relative to *act1*^+^. Error bars indicate mean ± SD (*n* = 3). (*D*) Measurement of LacZ expression driven by three 200-bp *cc2* fragments with promoter activity (cc2-200a, cc2-200b, and cc2-200c), the *nmt81* promoter, and no promoter following transformation of plasmids into wild type and *hap2*Δ using the ONPG substrate/absorbance at 420 nm. Error bars indicate mean ± SD (*n* = 3). (*Inset*) Diagram of plasmids with different 200-bp fragments from central domain region of *cen2* (cc2) placed upstream of the LacZ reporter. Significance of the differences observed between wild type and *hap2*Δ was evaluated using Student's *t*-test in *B*–*D*. (*) *P* < 0.05; (**) *P* < 0.005; (n.s.) not significant.

The central CENP-A^Cnp1^ domain of *S. pombe* centromeres is transcribed from many TSS and the resulting RNAs are short-lived (Choi et al. 2011; Sadeghi et al. 2014; Catania et al. 2015). Transcription can provide the opportunity for histone exchange/remodeling of resident nucleosomes (Venkatesh and Workman 2015). It is therefore feasible that transcription-coupled processes also promote the exchange of H3 for CENP-A^Cnp1^ in chromatin assembled on centromere DNA. Relatively high levels of RNAPII are detected on central domain DNA when assembled in H3 chromatin at ectopic *ura4:cc2* or on the pcc2 minichromosome, yet only low levels of RNAPII are detectable within endogenous centromeric central domain CENP-A^Cnp1^ chromatin (Catania et al. 2015; Shukla et al. 2018). Since CENP-A^Cnp1^ is primarily deposited during G2, we investigated whether Hap2 affects transcription from ectopic *ura4:cc2* in mid-G2 cells from *cdc25-22* synchronized cultures (Fig. 6A). qChIP was used to measure the levels of total RNAPII, initiating RNAPIIS5P and elongating RNAPIIS2P levels across *ura4:cc2*. RNAPIIS5P levels were significantly lower across this ectopic central domain DNA in *hap2*Δ compared with wild-type cells, but no obvious difference was detected within the CENP-A^Cnp1^ chromatin regions of endogenous centromeres (Fig. 6B). These data suggest that Hap2 promotes efficient transcriptional initiation from central domain DNA assembled in H3 chromatin (*ura4:cc2*). In contrast, the levels of total and elongating RNAPIIS2P associated with ectopic central domain DNA remained unchanged in *hap2*Δ relative to wild-type cells (Supplemental Fig. S6A,B). Consistent with reduced transcriptional initiation, lower levels of central domain transcripts were produced from ectopic *ura4:cc2* in *hap2*Δ cells (Fig. 6C). Transcript levels from outer repeats and *act1*^+^ were unaffected. Thus, Hap2 is required for efficient transcriptional initiation and transcript production from ectopic central domain DNA assembled in H3 chromatin. The fact that no decrease in elongating RNAPIIS2P was detected across this ectopic central domain in *hap2*Δ cells suggests that RNAPII remains associated with the central domain template for longer when Hap2 is absent.

Regions upstream of TSS within the central domain of *cen2* exhibit promoter activity (Catania et al. 2015). To determine whether Hap2 affects the transcription from these central domain promoters, the production of β-galactosidase was assessed when 200-bp promoter-containing cc2 fragments were placed upstream of lacZ (Fig. 6D, inset). Three central domain promoters exhibited significantly lower promoter activity in *hap2*Δ compared with wild-type cells, whereas the control *nmt81* promoter was unaffected (Fig. 6D; Supplemental Fig. S6C,D). We conclude that Hap2 is required for efficient transcription from central domain promoters and that this facilitates transcription-coupled histone exchange, thereby providing an opportunity for replacement of H3 with CENP-A^Cnp1^ to establish CENP-A^Cnp1^ chromatin domains and assemble functional kinetochores.

## Discussion

**Figure 7.**
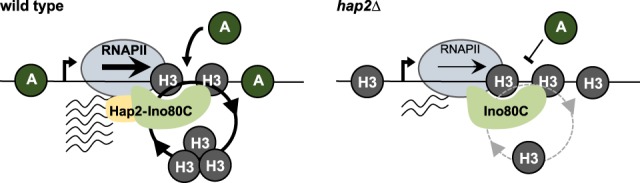
Hap2–Ino80-mediated transcription facilitates de novo establishment of CENP-A^Cnp1^ chromatin. Model for the establishment of CENP-A^Cnp1^ chromatin in *S. pombe*. (*Left*) Hap2 is required for transcription initiation events to maintain high H3 turnover thus renders H3 nucleosomes unstable on centromeric sequences allowing its replacement with CENP-A^Cnp1^ nucleosomes. (*Right*) Cells lacking Hap2 have lower transcription initiation events that stabilize the H3 nucleosomes on centromeric sequences thus lowering its propensity to establish CENP-A^Cnp1^ chromatin.

The mechanisms that contribute to the maintenance, and especially the establishment, of CENP-A chromatin remain poorly understood. To gain insight into how CENP-A chromatin is established on naïve centromere DNA, we applied proteomics to identify proteins associated with fission yeast CENP-A^Cnp1^ chromatin. In addition to kinetochore proteins, all Ino80C subunits, including the small auxiliary subunit, Hap2, were found to be significantly enriched in solubilized CENP-A^Cnp1^ chromatin. Hap2 and the Arp5 Ino80C subunit exhibit similar chromosomal distributions and Hap2 chromatin association depends on the Ies4 Ino80C subunit. Hap2 was found to promote CENP-A^Cnp1^ chromatin integrity at centromeres and to be required for the de novo establishment of CENP-A^Cnp1^ chromatin on introduced naïve centromere DNA. The requirement for Hap2 in ensuring high histone H3 turnover on endogenous centromere DNA, ectopically located centromere DNA, and noncentromeric CENP-A^Cnp1^ islands indicates that Hap2–Ino80C drives H3 nucleosome turnover at these locations. The loss of CENP-A^Cnp1^ incorporation from NCIS islands in the absence of Hap2 underscores the role for Hap2–Ino80C-mediated H3 turnover in stimulating CENP-A^Cnp1^ incorporation. Strikingly, Hap2 is required for efficient transcription specifically from central domain promoters. We propose a mechanism in which Hap2–Ino80C drives the inherent instability of H3 nucleosomes on centromeric DNA via transcription-coupled nucleosome turnover, providing the opportunity for incorporation of CENP-A^Cnp1^ in place of histone H3, when CENP-A^Cnp1^ is available (Fig. 7).

Studies in a variety of species have shown that Ino80C influences transcription (Cai et al. 2007; Klopf et al. 2009; Hogan et al. 2010). The noncanonical human Yin Yang 1 (YY1) transcription factor is known to associate with Ino80 and facilitate both transcriptional activation and repression (Yao et al. 2001; Cai et al. 2007). It is also known that Ino80 suppresses antisense and other noncoding transcription (Alcid and Tsukiyama 2014; Marquardt et al. 2014). The noncoding transcription of centromeric DNA by RNAPII has been implicated in CENP-A deposition in several systems (Chueh et al. 2009; Chan et al. 2012; Rošić et al. 2014; Grenfell et al. 2016; McNulty et al. 2017). However, both reduced and increased transcription of centromere DNA appears to be incompatible with CENP-A chromatin integrity and centromere function (Hill and Bloom 1987; Nakano et al. 2008; Ohkuni and Kitagawa 2011; Bergmann et al. 2012). Such observations suggest that an appropriate level and type of programmed transcription may be required to promote CENP-A assembly on centromere DNA.

Each fission yeast centromere contains a central domain of ∼10 kb assembled in CENP-A^Cnp1^ rather than H3 nucleosomes. Multiple transcriptional start sites are detected on both strands when central domain is assembled in H3, rather than CENP-A^Cnp1^, chromatin at an ectopic locus (Choi et al. 2011; Catania et al. 2015), indicating that these regions are pervasively transcribed. Distinct Ino80C modules undertake particular tasks such as nucleosome binding, sliding, and ATPase activity (Chen et al. 2011). The specific impact of Hap2 on Ino80C activity and how this is altered by loss of other subunits such as Ies4 remains to be determined. However, a major activity of Ino80C is to slide nucleosomes relative to sizable lengths of flanking unoccupied DNA (Udugama et al. 2011). Thus, the generation of nucleosome free regions that facilitate RNAPII recruitment, and consequently transcription, are an intrinsic facet of Ino80C function. Consequently, the loss of Hap2–Ino80C is expected to occlude central domain promoters with nucleosomes and result in reduced transcriptional initiation.

Since RNAPII recruitment to central domain chromatin during G2 phase of the cell cycle is coincident with H3 eviction and CENP-A^Cnp1^ incorporation (Shukla et al. 2018), it is likely that these events are somehow coupled. The conserved heptad repeat composing the C-terminal domain (CTD) of RNAPII undergoes S5 phosphorylation (S5P) at promoters upon transcription initiation and S2 phosphorylation (S2P) in coding regions during transcriptional elongation (Komarnitsky et al. 2000). RNAPII stalls when it encounters obstacles such as DNA damage or natural barriers (Poli et al. 2017). Despite relatively high levels of RNAPII being detected on H3-assembled ectopic central domain, meagre levels of transcripts are produced, consistent with transcriptional stalling (Choi et al. 2011; Catania et al. 2015). Analyses in *S. cerevisiae* show that Ino80 is required to release stalled RNAPII from chromatin and enable its proteasomal degradation (Lafon et al. 2015). Previously, we showed that mutants (*ubp3*Δ, *tfs1*Δ) expected to increase the levels of stalled RNAPIIS2P on central domain chromatin, promote CENP-A incorporation (Catania et al. 2015). Interestingly, cells lacking Hap2 display lower initiating RNAPIIS5P over ectopic H3-assembled central domain chromatin in G2 but the levels of total RNAPII and elongating RNAPII-S2P are unaltered (Fig. 6B; Supplemental Fig. S6A,B). This indicates that, although lower levels of initiation and transcription take place within ectopic central domain chromatin in the absence of Hap2, elongating RNAPII is retained across the domain. We interpret these observations to indicate that Hap2–Ino80C is required to release elongating RNAPII that becomes trapped or stalled in central domain chromatin. We suggest that resident H3 nucleosomes are evicted by Hap2–Ino80C as part of the process involved in removing this stalled RNAPII. Thus, centromere DNA may be programmed to be pervasively transcribed and stall RNAPII over a relatively large 10-kb region in order to recruit Hap2–Ino80C and trigger H3 turnover throughout this extensive domain, thereby providing the opportunity for CENP-A incorporation. Stalling may result from collisions between converging RNAPII complexes or other obstacles such as the replication origin recognition complex (ORC) which is bound to the many AT-rich tracts present within central domain regions (Hayashi et al. 2007). Alternatively, elongating RNAPII on central domain DNA may be somehow earmarked for removal using cues analogous to those that allow Ino80 to suppresses antisense and cryptic unstable transcripts (Alcid and Tsukiyama 2014). Thus, pervasive transcription across an extensive region such as the central domain may itself provoke Ino80C recruitment.

Histone turnover rates are generally higher for nucleosomes close to promoters (Dion et al. 2007). The +1 nucleosomes exhibit high levels of turnover and Ino80C has been reported to mediate the exchange of H2A.Z for canonical H2A in such nucleosomes (Papamichos-Chronakis et al. 2011; Watanabe et al. 2013; Yen et al. 2013). However, H2A.Z turnover on transcribed genes has been shown to be not reliant on Ino80 activity (Tramantano et al. 2016). Moreover, Ino80 is also known to reduce transcription from some promoters, independently of its role in removing H2A.Z (Barbaric et al. 2007). The fact that no difference in the levels of H2A.Z^Pht1^ was detected across the ectopic central domain in *hap2*Δ cells relative to wild-type (Supplemental Fig. S5B) suggests that Hap2–Ino80C does not mediate H2A.Z^Pht1^ eviction from central domain chromatin and that it must affect some other aspect of centromere promoter function, perhaps through its known nucleosome sliding activity. Thus, Ino80C may be required to slide nucleosomes away from central domain promoters so that they are efficiently transcribed and the resulting transcriptional properties of this domain mediate H3 nucleosome turnover.

Previously, we showed that CENP-A^Cnp1^ competes with histone H3 for incorporation into centromeric chromatin and CENP-A^Cnp1^ incorporation is promiscuous, replacing H3 when the opportunity arises (Castillo et al. 2007; Choi et al. 2011, 2012). Thus, high histone H3 nucleosome turnover, or low nucleosome occupancy, appears to be an underlying property of many sequences that are prone to CENP-A^Cnp1^ assembly in fission yeast. As transcription appears to be widespread at centromeres in other organisms, high turnover of resident H3 nucleosomes, stimulated by Ino80C, may be a conserved attribute of centromeric DNA that stimulates CENP-A deposition.

## Materials and methods

Additional methods are described in the Supplemental Material, including lists of strains (Supplemental Table S6), plasmids (Supplemental Table S7), and primers (Supplemental Table S8) used in this study.

### Affinity purification mass spectrometry analysis

Native coimmunoprecipitated samples were analyzed on an Orbitrap Fusion Lumos Tribrid mass spectrometer and on a Q Exactive (both from Thermo Fisher Scientific) both coupled online to an Ultimate 3000 RSLCnano system (Dionex, Thermo Fisher Scientific). For details of the separation, mass spectrometer parameters, and data analysis, see the Supplemental Material and Supplemental Tables S3–S5. The MS proteomics data have been deposited to the ProteomeXchange Consortium via the Proteomics Identifications (PRIDE) (Vizcaíno et al. 2016) partner repository with the data set identifier PXD016602.

### Establishment assays

Cells transformed with minichromosomes were plated on PMG-uracil-adenine plates and incubated for 5–10 d at 32°C until medium-sized colonies had grown. Colonies were replica-plated to PMG low-adenine (10 µg/mL) plates to determine the frequency of establishment of centromere function. These indicator plates allow minichromosome loss (red colony) or retention (white/pale pink colony) to be determined. In the absence of centromere establishment, minichromosomes behave as episomes that are rapidly lost. Minichromosomes occasionally integrate giving a false-positive white phenotype. To assess the frequency of such integration events and to confirm establishment of centromere segregation function, colonies giving the white/pale-pink phenotype upon replica plating were restreaked to single colonies on low-adenine plates. Sectored colonies are indicative of segregation function with low levels of minichromosome loss, whereas pure white colonies are indicative of integration into endogenous chromosomes and the establishment frequency was adjusted accordingly.

### LacZ assays

LacZ assays were performed as described (Guarente 1983). Plasmids containing LacZ with upstream nmt81 promoter, 200-bp sequences from centromere 2 or no promoter were used as described (Catania et al. 2015). Plasmids were transformed into wild-type and *hap2*Δ strains and grown on minimal medium.

### ChIP-seq and ChIP-nexus

ChIP-seq and ChIP-nexus were prepared essentially as described (Shukla et al. 2018). For details, see the Supplemental Material. The accession number of the sequencing data reported for Hap2-GFP and Arp5-3HA is GEO GSE141524 and for CENP-A^Cnp1^ (HA and T7 tag), GEO GSE136305.

## Supplementary Material

Supplemental Material
