# Supplementary material for: Hap2–Ino80-facilitated transcription promotes de novo establishment of CENP-A chromatin
Source: Genes Dev. 2020 Feb 1;34(3-4):226–38. doi: 10.1101/gad.332536.119 (PMC7000912; doi:10.1101/gad.332536.119)
Supplement: Supplemental Material [file supp_34_3-4_226__index.html]

Hap2–Ino80-facilitated transcription promotes de novo establishment of CENP-A chromatin — Supplemental Material 

# Hap2–Ino80-facilitated transcription promotes de novo establishment of CENP-A chromatin

## Supplemental Material

- Supplemental\_Table\_S7.xlsx
- Supplemental\_Table\_S5.xlsx
- Supplemental\_Table\_S3.xlsx
- Supplemental\_Table\_S1.xlsx
- Supplemental\_Table\_S8.xlsx
- Supplemental\_Table\_S6.xlsx
- Supplemental\_Table\_S4.xlsx
- Supplemental\_Table\_S2.xlsx
- Supplemental\_Material.pdf
