# Supplementary material for: Hap2–Ino80-facilitated transcription promotes de novo establishment of CENP-A chromatin
Source: Genes Dev. 2020 Feb 1;34(3-4):226–38. doi: 10.1101/gad.332536.119 (PMC7000912; doi:10.1101/gad.332536.119)
Supplement: Supplemental Material [file supp_gad.332536.119_Supplemental_Material.pdf]

Hap2-Ino80 facilitated transcription promotes de novo establishment of CENP-A chromatin

Puneet P. Singh, Manu Shukla, Sharon A. White, Marcel Lafos, Pin Tong, Tatsiana Auchynnikava, Christos Spanos, Juri Rappsilber, Alison L. Pidoux and Robin C. Allshire

Supplementary Table Titles

Table S1. Compositions of *S. pombe*, *S. cerevisiae* and *H. sapiens* Ino80-related chromatin remodeling complex

Table S2. Orthologous *S. pombe*, *S. cerevisiae* and *H. sapiens* kinetochore proteins

Table S3. AP-MS analysis of CENP-A^{Cnp1} chromatin

Table S4. AP-MS analysis of Hap2-GFP

Table S5. AP-MS analysis of Ino80-3HA

Table S6. List of strains used in the study

Table S7. List of plasmids used in the study

Table S8. List of primers used in the study

Supplementary Materials and Methods

Cell growth and manipulation

Standard genetic and molecular techniques were followed. Fission yeast methods were as described (Moreno et al. 1991). Fission yeast strains are listed in Table S3. YES (Yeast Extract with Supplements) was used as a rich medium or PMG (Pombe Minimal Glutamate) for growth of cells in liquid cultures. 4X-YES was used for experiments where higher cell numbers were required. H3.2 RITE strains containing HA/T7 RITE cassettes were as described (Verzijlbergen et al. 2010; Shukla et al. 2018). Plasmids used in this study are listed in Table S4.

Native immunoaffinity purification

Cell were grown in 4X-YES and 2-4×10^{10} cells were used for immunoprecipitation. Cells were harvested by centrifugation at 3500×g, washed twice with water and flash frozen in liquid nitrogen. Frozen cell pellets were ground using Retsch MM400 mill. The grindate was resuspended in lyisis buffer (10 mM Tris pH7.4, 5 mM CaCl2, 5 mM MgCl2, 50 mM NaCl, 0.1% IGEPAL-CA630 and supplemented with protease inhibitor (P8215, Sigma) and 2 mM PMSF and thawed for 30 minutes on ice. Chromatin was solubilized by incubation with 4-20 units of Micrococcal nuclease (N3755, Sigma) for 10 minutes at 37° C. MNase digestion was stopped by adding EGTA to 20 mM and lysates were rotated at 4° C for 1 hour to ensure chromatin solubilization. Supernatant was separated from lysates by centrifugation at 20,000×g for 10 minutes and used for immunoprecipitation using 20 µg of anti-GFP antibody (11814460001, Roche) (used for GFP-CENP-A^{Cnp1} and Hap2-GFP IP) or 20 µg of anti-HA antibody (Roche, ROAHAHA) (used for Ino80-3HA IP) coupled to 50 µl of Protein G Dynabeads (Life Technologies) using dimethyl pimelimidate (DMP; 21666, ThermoFisher Scientific). Bead-bound affinity-selected proteins were washed three times with lysis buffer.

Elution was performed with 0.1% RapiGest SF (186001860, Waters) in 50mM Tris-HCl pH8 by incubating at 50° C for 10 minutes.

Co-immunoprecipitation

The co-immunoprecipitation protocol is similar to native immunoaffinity purification described above with following modifications. Cell were grown in 4X-YES and 2×10^{10} cells were flash frozen in liquid nitrogen. Frozen cell pellets were ground using Retsch MM400 mill. The grindate was resuspended in lyisis buffer (10 mM Tris pH7.4, 5 mM $CaCl_2$, 5 mM $MgCl_2$, 50 mM NaCl, 500mM KCl 0.1% IGEPAL-CA630 and supplemented with protease inhibitor (P8215, Sigma) and 2 mM PMSF and thawed for 30 minutes on ice. Chromatin was solubilized by incubation with 100 units of Micrococcal nuclease (Sigma, N3755) for 20 minutes at 37° C. Solubilized chromatin was further digested with 1000 units of Benzonase (Sigma, E1014) or incubated in 100 µg/ml ethidium bromide. Immunoprecipitation was performed using 20 µg of anti-GFP antibody (Roche, 11814460001) (used for Hap2-GFP IP) or 20 µg of anti-HA antibody (Roche, ROAHAHA) (used for Ino80-3HA IP) coupled to 25 µl of Protein G Dynabeads (Life Technologies) using DMP. Bead-bound affinity-selected proteins were washed five times with lysis buffer for 5 minute and eluted with LDS loading buffer (ThermoFisher Scientific, 84788) by incubating at 75° C for 10 minutes. Western blotting detection was performed using anti-GFP (ThermoFisher Scientific, A11122) or anti-HA antibody (Roche, ROAHAHA) and HRP conjugated anti-Rabbit IgG HRP (Sigma, A6154)).

Mass spectrometry and analysis

Proteins were digested with trypsin into peptides and analyzed by mass spectrometry. Proteins were denatured by adding final 25mM DTT to the immunoprecipitated proteins and incubated at 95° C for 5 minutes. Samples were cooled at room

temperature and 200 µl of 8M Urea in 100mM Tris-HCl pH8 was added and passed through Vivacon 500 column (VN01H21, Sartorius Vivacon 500, 30,000 MWCO Hydrosart) by centrifugation at 14,000xg for 30 minutes. Cysteine residues were alkylated to prevent free sulfhydryls from reforming disulfide bonds by incubation with 100 µl of 0.05 M Iodoacetamide (Sigma) in 8 M urea for 20 minutes at 27° C in the dark and centrifuged at 14,000xg to remove the supernatant. Columns were then washed once with 100 µl of 8M urea and twice with 100 µl of 0.05 M ammonium bicarbonate (Sigma). Proteins were digested by addition of 100 µl of 0.05 M ABC containing 0.3 µg of trypsin (90057, Thermo Scientific) at 37° C for 16 hours. Columns were spun at 14,000xg to collect the digested peptides and washed again with 100 µl of 0.05 M ABC. Trypsinization was stopped by addition of 10 µl of 10% trifluoroacetic acid (TFA) to bring the pH to below 2.

C18 reverse-phase resin (66883-U, Sigma) was used to desalt peptide samples prior to LC-MS/MS. StageTips were packed tightly with 2 layers of C18 resin. The resin was conditioned with 30 µl of 100% methanol, washed with 30 µl of 80% acetonitrile to remove impurities and finally equilibrated by passing 30 µl of 0.1% TFA. The trypsinized peptide solution was passed through the stage tip by centrifugation at 2600 rpm for binding. Following stage tip desalting, samples were diluted with equal volume of 0.1% TFA and spun onto StageTips as described (Rappsilber et al., 2003). Peptides were eluted in 40 µL of 80% acetonitrile in 0.1% TFA and concentrated down to 2 µL by vacuum centrifugation (Concentrator 5301, Eppendorf, UK). The peptide sample was then prepared for LC-MS/MS analysis by diluting it to 5 µL by 0.1% TFA.

LC-MS-analyses were performed on an Orbitrap Fusion™ Lumos™ Tribrid™ Mass Spectrometer and on a Q Exactive (both from Thermo Fisher Scientific, UK) both coupled on-line, to an Ultimate 3000 RSLCnano Systems (Dionex, Thermo Fisher

Scientific, UK). In both cases, peptides were separated on a 50 cm EASY-Spray column (Thermo Scientific, UK), which was assembled on an EASY-Spray source (Thermo Scientific, UK) and operated at 50° C. Mobile phase A consisted of 0.1% formic acid in LC-MS grade water and mobile phase B consisted of 80% acetonitrile and 0.1% formic acid. Peptides were loaded onto the column at a flow rate of 0.3 μL min^{-1} and eluted at a flow rate of 0.2 μL min^{-1} according to the following gradient: 2 to 40% mobile phase B in 150 min and then to 95% in 11 min. Mobile phase B was retained at 95% for 5 min and returned back to 2% a minute after until the end of the run (190 min).

For Fusion Lumos, FTMS spectra were recorded at 120,000 resolution (scan range 350-1500 m/z) with an ion target of 7.0×10^5. MS2 was performed in the ion trap with ion target of 1.0×10^4 and HCD fragmentation (Olsen et al. 2007) with normalized collision energy of 27. The isolation window in the quadrupole was 1.4 Thomson. Only ions with charge between 2 and 7 were selected for MS2. For Q Exactive, FTMS spectra were recorded at 70,000 resolution (scan range 350-1400 m/z) and the ten most intense peaks with charge ≥ 2 of the MS scan were selected with an isolation window of 2.0 Thomson for MS2 (filling 1.0×10^6 ions for MS scan, 5.0×10^4 ions for MS2, maximum fill time 60 ms, dynamic exclusion for 50 s).

The MaxQuant software platform (Cox and Mann 2008) version 1.5.2.8 was used to process the raw files and search was conducted against *Schizosaccaromyces pombe* complete/reference proteome set of PomBase database (released in July, 2015), using the Andromeda search engine (Cox et al. 2011). For the first search, peptide tolerance was set to 20 ppm while for the main search peptide tolerance was set to 4.5 pm. Isotope mass tolerance was 2 ppm and maximum charge to 7. Digestion mode was set to specific with trypsin allowing maximum of two missed cleavages.

Carbamidomethylation of cysteine was set as fixed modification. Oxidation of methionine, phosphorylation of serine, threonine and tyrosine and ubiquitination of lysine were set as variable modifications. Label-free quantitation analysis was performed by employing the MaxLFQ algorithm as described by Cox et al. 2014. Absolute protein quantification was performed as described in Schwanhüusser et al. 2011. Peptide and protein identifications were filtered to 1% FDR.

The Perseus software platform (Tyanova et al. 2016) version 1.5.5.1 was used to process LFQ intensities of the proteins generated by MaxQuant. The LFQ intensities were transformed to log2 scale and list was filtered for proteins with at least 2-3 valid values in any sample. Missing values were imputed from normal distribution for that IP.

Cytology

Cells were fixed with 3.7% formaldehyde (Sigma) for 7 minutes at room temperature. Immunolocalization staining was performed as described (Shukla et al. 2018). The following antibodies were used at 1:100 dilution: Anti-CENP-A^{Cnp1} (sheep in-house), anti-GFP (ThermoFisher Scientific, A11122), anti-TAT1 (mouse in-house); Alexa 594 and 488 labelled secondary antibodies at 1:1000 dilution (Life Technologies). Images were acquired with Zeiss LSM 880 confocal microscope equipped with Airyscan superresolution imaging module, using a 100X/1.40 NA Plan-Apochromat Oil DIC M27 objective lens and processed using ZEN Black image acquisition and processing software (Zeiss MicroImaging).

LacZ assay

LacZ assay was performed as described (Guarente 1983). Plasmids containing LacZ with upstream nmt81 promoter, 200 bp sequences from centromere 2 or no promoter

were used as described (Catania et al. 2015). Plasmids were transformed into wild-type and *hap2*Δ strains and grown on minimal medium.

ChIP-qPCR

Cells were grown at appropriate temperature and medium. Cultures were fixed with 3.7% formaldehyde for 15 min at room temperature. ChIP was performed as previously described (Castillo et al. 2007). Cells were lysed by bead-beating (Biospec Products) and sonicated in a Bioruptor (Diagenode) (20 min, 30 s On and 30 s Off at 'High' (200 W). 10 µl anti-CENP-A^{Cnp1} (sheep in-house antiserum) and 25 µl Protein-G-Agarose beads (Roche) or 2 µl of anti-GFP (A11122, ThermoFisher Scientific), 2 µl of anti-H3 (ab1791, Abcam), 2 µl of anti-T7 (69522, Millipore), 2 µl of anti-myc (2276, Cell Signaling Technology), 1 µl of anti-H3K9me (m5.1.1), 2 µl of anti-RNAPII (ab817, Abcam), 2 µl of RNAPII S2P (Abcam, ab5095) and 2 µl of RNAPII S5P (61085, Actif motif) and 20 µl Protein-G-Dyna beads (Life Technologies) were used per ChIP. qPCR was performed using Light Cycler 480 SybrGreen Master Mix (Roche, 04887352001) and analysed using Light Cycler 480 Software 1.5 (Roche). Primers used in qPCR are listed in Supplementary Table S5.

qRT-PCR

RNA was isolated with RNeasy mini kit (Qiagen) according to the manufacturer's protocol. A total of 10 µg RNA isolated by RNeasy Mini kit (Qiagen) was treated with 1 µl of Turbo DNase (AM2238, Invitrogen) for 30 minutes at 37°C. A second digestion was followed by adding additional 1 µl of Turbo DNase for 30 minutes at 37°C. RNA was then cleaned up according to RNeasy Mini Protocol for RNA Cleanup (Qiagen) and dissolved in nuclease free water. For qRT-PCR analysis, first strand cDNA synthesis was performed using 100 ng of random hexamer (Invitrogen), 1 µg of DNA-free RNA template and 1 µl of Superscript III (Invitrogen) reverse transcriptase

according to the manufacturer's instruction. As a negative control (-RT), the same reaction was performed without SuperScript III.

ChIP-Seq

ChIP and library preparation was performed essentially as described (Shukla et al. 2018). Quality filtering was performed using trimmomatic v0.36. Read mapping was performed using bwa v0.7.17 to *S. pombe* genome GCF_000002945.1. Bigwigs were generated using bamCompare v3.1.3 from deeptools. Plot in Fig. 2F was generated using plotHeatmap from deaptools. Peak calling was performed using macs2 v2.1.2. Overlap of the peak files were called using bamtools v2.5.1.

ChIP-Nexus

ChIP-Nexus was prepared essentially as described (Shukla et al. 2018). Cells were grown in 4X-YES at 32°C. Briefly, cell pellets corresponding to $7.5X10^{8}$ cells were lysed by four 1 minute cycles of bead beating in 500 µl of lysis buffer (50 mM HEPES-KOH, pH 7.5, 140 mM NaCl, 1 mM EDTA, 1% Triton X-100, 0.1% sodium deoxycholate). Insoluble chromatin fraction was isolated by centrifugation at 6000×g. The pellet was washed with 1 ml lysis buffer and gently resuspended in 300 µl lysis buffer containing 0.2% SDS and sheared by sonication with Bioruptor (Diagenode) for 30 minutes (30s On, 30s off at high setting). 900 µl of lysis buffer (without SDS) was added and samples were clarified by centrifugation at 17000×g for 20 minutes. Supernatants were used for ChIP. Supernatants were incubated with 6 µl anti-HA (12CA5, in-house preparation) or 6 µl anti-T7 (69522, Millipore) and protein G-dynabeads (ThermoFisher Scientific) for ChIP.

ChIP-Nexus libraries were prepared essentially as described (He et al. 2015). DNA was end repaired using T4 DNA polymerase (NEB, M0203S), DNA polymerase I large fragment (NEB, M0210S) and T4 polynucleotide kinase (NEB, M0201S). A single 3'-A

overhang was added using Klenow exo- polymerase. Adapters were ligated and blunted again by Klenow exo- polymerase to fill in the 5' overhang first and then by T4 DNA polymerase to trim possible 3' overhangs. Blunted DNA was then sequentially digested by lambda exonuclease (NEB, M0262S) and RecJf (NEB, M0264L). Digested single strand DNA was then eluted, reverse cross-linked and phenol-chloroform extracted. Fragments were then self-circularized by Circligase (Epicentre, CL4111K). An oligonucleotide was hybridized to circularized single DNA for subsequent BamHI digestion in order to linearize the DNA. This linearized single strand DNA was then PCR-amplified using adapter sequences and libraries were purified and size selected using Ampure XP beads. The libraries were sequenced following Illumina HiSeq2500 work flow.

More than 20 million 50bp long single end reads were generated for each sample in this experiment. All reads first 15bp were trimmed, then aligned on S.pombe v2.20 genome build with bowtie2 (Langmead and Salzberg 2012). Alignment analysis were used by samtools (Li et al. 2009) and bedtools (Quinlan and Hall 2010). Genome wide enrichment visualization was use deeptools (Ramírez et al. 2014) and IGV browser. ChIP peak were called by MACE 1.2 (Wang et al. 2014).

Supplemental References

Castillo AG, Mellonea BG, Partridgeb JF, Richardsonc W, Hamilton GL, Allshire RC, Pidoux AL. 2007. Plasticity of fission yeast CENP-A chromatin driven by relative levels of histone H3 and H4. *PLoS Genet* **3:** e121.

Catania S, Pidoux AL, Allshire RC. 2015. Sequence Features and Transcriptional Stalling within Centromere DNA Promote Establishment of CENP-A Chromatin

ed. B. Van Steensel. *PLoS Genet* **11**: e1004986.

Cox J, Hein MY, Luber CA, Paron I, Nagaraj N, Mann M. 2014. Accurate proteome-wide label-free quantification by delayed normalization and maximal peptide ratio extraction, termed MaxLFQ. *Mol Cell Proteomics* **13:** 2513-26.

Cox J, Mann M. 2008. MaxQuant enables high peptide identification rates, individualized p.p.b.-range mass accuracies and proteome-wide protein quantification. *Nat Biotechnol* **26:** 1367-72.

Cox J, Neuhauser N, Michalski A, Scheltema RA, Olsen J V., Mann M. 2011. Andromeda: A peptide search engine integrated into the MaxQuant environment. *J Proteome Res* **10:** 1794-805.

Guarente L. 1983. Yeast Promoters and lacZ Fusions Designed to Study Expression of Cloned Genes in Yeast. *Methods Enzymol* **101:** 181-91.

He Q, Johnston J, Zeitlinger J. 2015. ChIP-nexus enables improved detection of in vivo transcription factor binding footprints. *Nat Biotechnol* **33:** 395-401.

Langmead B, Salzberg SL. 2012. Fast gapped-read alignment with Bowtie 2. *Nat Methods* **9:** 357-9.

Li H, Handsaker B, Wysoker A, Fennell T, Ruan J, Homer N, Marth G, Abecasis G, Durbin R. 2009. The Sequence Alignment/Map format and SAMtools. *Bioinformatics* **25:** 2078-9.

Moreno S, Klar A, Nurse P. 1991. Molecular genetic analysis of fission yeast Schizosaccharomyces pombe. *Methods Enzymol* **194:** 795-823.

Olsen J V., Macek B, Lange O, Makarov A, Horning S, Mann M. 2007. Higher-energy C-trap dissociation for peptide modification analysis. *Nat Methods* **4:** 709-12.

Quinlan AR, Hall IM. 2010. BEDTools: A flexible suite of utilities for comparing genomic features. *Bioinformatics* **26:** 841-2.

Ramírez F, Dündar F, Diehl S, Grüning BA, Manke T. 2014. DeepTools: A flexible platform for exploring deep-sequencing data. *Nucleic Acids Res* **42:** W187-91.

Schwanhüusser B, Busse D, Li N, Dittmar G, Schuchhardt J, Wolf J, Chen W, Selbach M. 2011. Global quantification of mammalian gene expression control. *Nature* **473:** 337-42.

Shukla M, Tong P, White SA, Singh PP, Reid AM, Catania S, Pidoux AL, Allshire RC. 2018. Centromere DNA Destabilizes H3 Nucleosomes to Promote CENP-A Deposition during the Cell Cycle. *Curr Biol* **28**: 3924-3936.e4.

Tyanova S, Temu T, Sinitcyn P, Carlson A, Hein MY, Geiger T, Mann M, Cox J. 2016. The Perseus computational platform for comprehensive analysis of (prote)omics data. *Nat Methods* **13:** 731-40.

Verzijlbergen KF, Menendez-Benito V, van Welsem T, van Deventer SJ, Lindstrom DL, Ovaa H, Neefjes J, Gottschling DE, van Leeuwen F. 2010. Recombination-induced tag exchange to track old and new proteins. *Proc Natl Acad Sci* **107:** 64-8.

Wang L, Chen J, Wang C, Uusküla-Reimand L, Chen K, Medina-Rivera A, Young EJ, Zimmermann MT, Yan H, Sun Z, et al. 2014. MACE: model based analysis of ChIP-exo. *Nucleic Acids Res* **42:** e156.

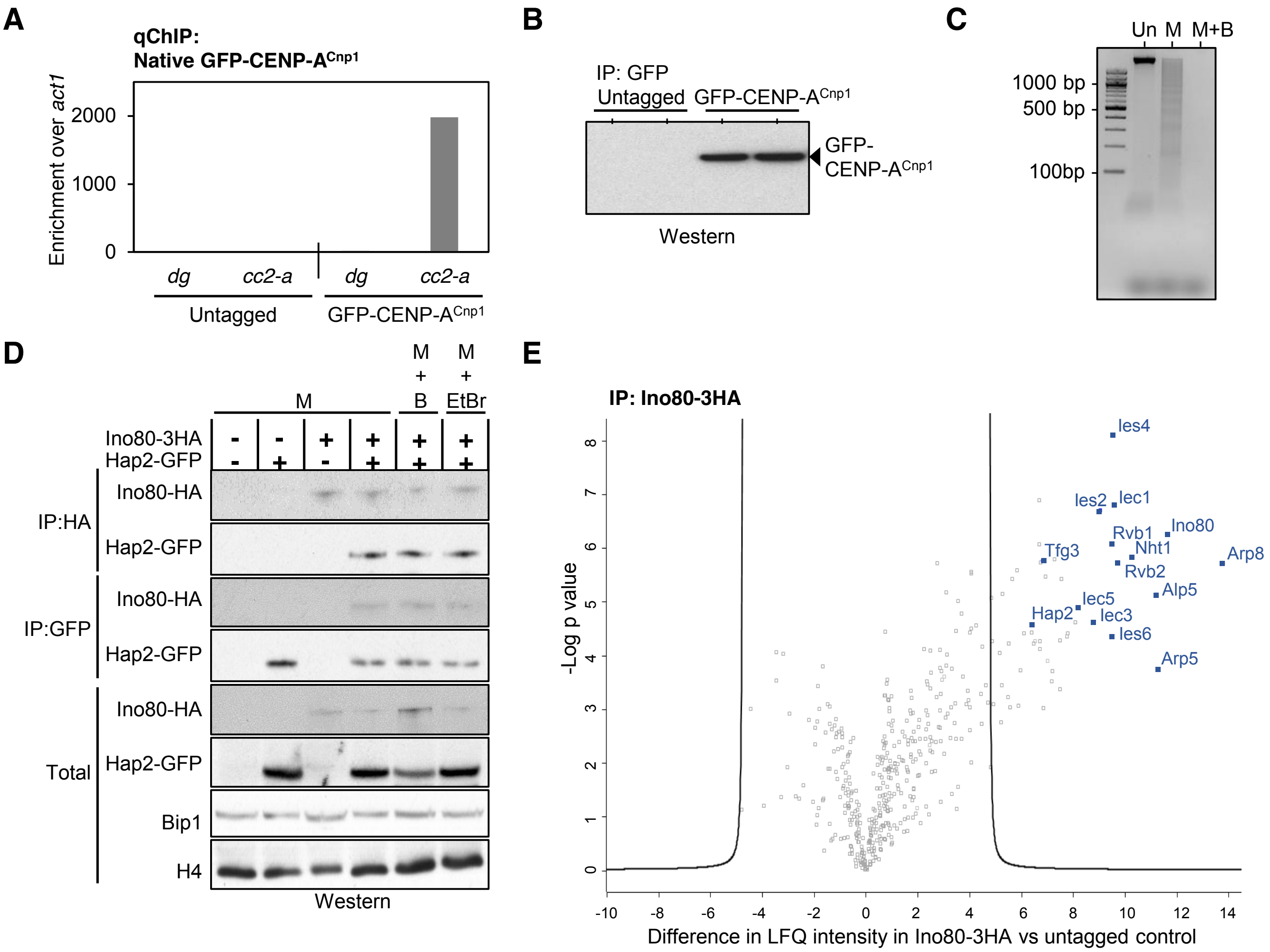

Figure S1. Purification of CENP-A^{Cnp1} and Ino80 complex. (A) Native qChIP for GFP-CENP-A^{Cnp1} at outer repeat heterochromatin (*dg*) and within endogenous centromeres (*cc2-a*). (B) Anti-GFP IP from lysates of cells expressing GFP-CENP-A^{Cnp1} or untagged control cells, followed by anti-GFP western analysis. (C) EtBr stained gel of DNA purified before and after nuclease digestion from samples used in (D). M: MNase ; M+B: MNase and Benzonase. (D) Western analysis of anti-HA IPs and anti-GFP IPs to enrich Ino80-3HA or Hap2-GFP, respectively, from lysates of cells expressing Ino80-3HA, Hap2-GFP or both, or untagged tagged control. Chromatin is digested with either MNase or Benzonase or both. MNase digested chromatin was also incubated in 100 µg/ml Ethidium bromide during IPs to prevent DNA-mediated interactions. Western analysis to detect Ino80-3HA or Hap2-GFP in anti-HA IPs, anti-GFP IPs and total extracts. Loading control: Bip1 and anti-histone H4. (E) Volcano plot comparing LFQ intensity of proteins enriched in affinity selected Ino80-3HA (anti-HA) versus untagged control. Ino80 complex subunits (Blue).

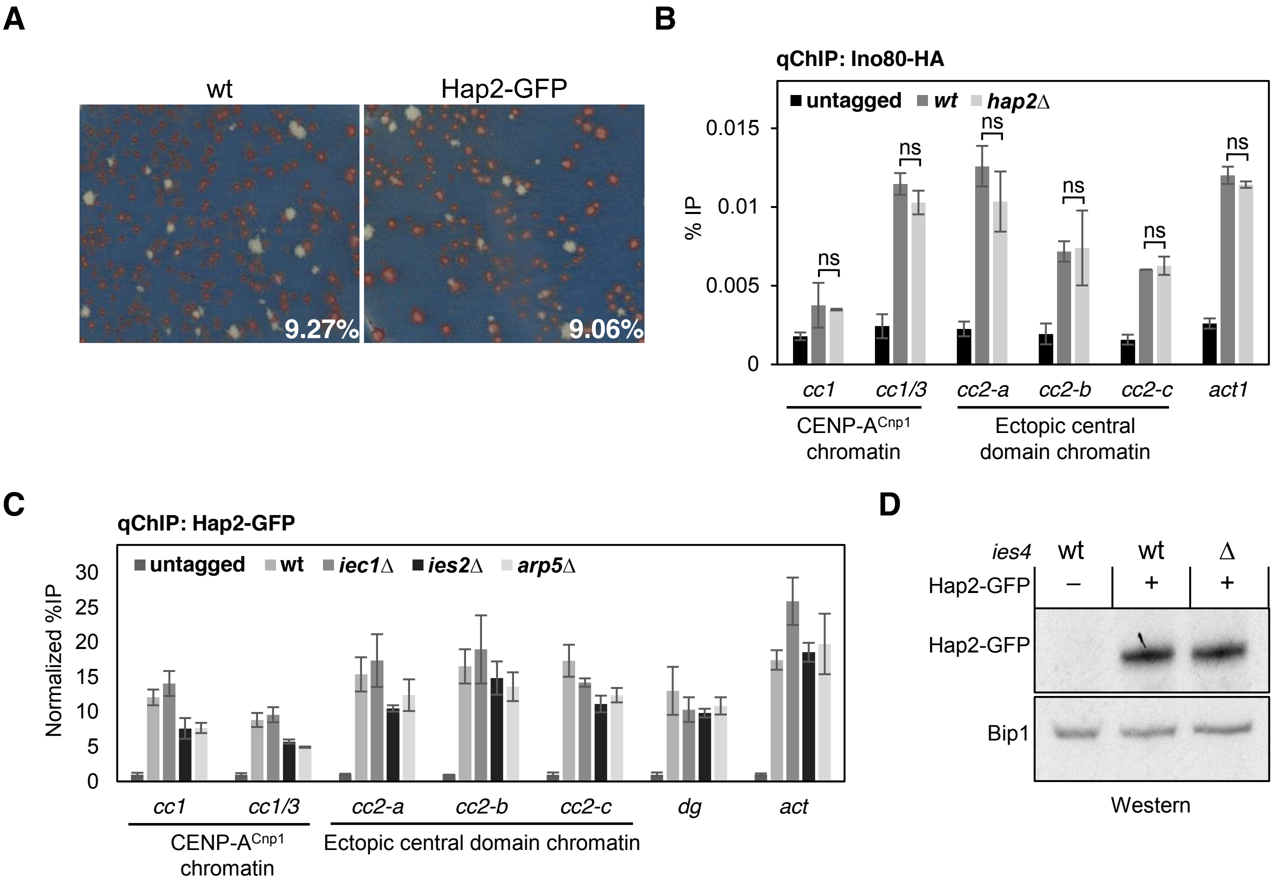
A
wt
Hap2-GFP
9.27%
9.06%
B
qChIP: Ino80-HA
untagged
wt
hap2Δ
% IP
0.015
0.01
0.005
0
ns
cc1
cc1/3
cc2-a
cc2-b
cc2-c
act1
CENP-ACnp1 chromatin
Ectopic central domain chromatin
C
qChIP: Hap2-GFP
untagged
wt
iec1Δ
ies2Δ
arp5Δ
Normalized %IP
30
25
20
15
10
5
0
cc1
cc1/3
cc2-a
cc2-b
cc2-c
dg
act
CENP-ACnp1 chromatin
Ectopic central domain chromatin
D
ies4
wt
wt
Δ
Hap2-GFP
–
+
+
Hap2-GFP
Bip1
Western

Figure S2. C-terminal tagging of Hap2 is functional. Ino80 localization at central domain chromatin is independent of Hap2. Hap2 chromatin localization is also independent of Iec1, Ies2 and Arp5. Loss of Ies4 does not affect cellular levels of Hap2-GFP. (A) *S. pombe* transformed with pHcc2 minichromosome DNA were replica-plated to low adenine non-selective plates. Colonies where CENP-A^{Cnp1} chromatin and functional centromeres have established retain pHcc2 and exhibit a white/pale-pink colour. Those that do not establish centromeres lose pHcc2 and form red colonies. Representative plates of such establishment assay are shown for wild type (n = 539) and Hap2-GFP (n = 342) cells. The percentage of transformants exhibiting functional centromere establishment are indicated. (B) qChIP for Ino80-HA at two locations within endogenous centromeres (*cc1* and cc1/3), three locations within ectopically inserted central domain 2 DNA (*cc2-a*, *cc2-b* and *cc2-c*) and non-centromere locus (*act1⁺*), in wild type and *hap2*Δ. Error bars indicate mean ± SD (n = 3). Significance of the differences observed between wild type and *hap2*Δ was evaluated using Student's t-test; * $p < 0.05$, ** $p < 0.005$; *** $p < 0.005$ and n.s., not significant. (C) qChIP for Hap2-GFP at three locations within ectopic central domain H3 chromatin (*cc2-a*, *cc2-b* and *cc2-c*), endogenous centromeres (*cc1* and *cc1/3*), outer repeat heterochromatin (*dg*) and non-centromere locus (*act1⁺*) in untagged, wild-type, *iec1*Δ*, ies2*Δ *and arp5*Δ cells. (D) Western analysis of Hap2-GFP and Bip1 (loading control) in whole cell lysates of wild-type and *ies4*Δ cells expressing GFP-CENP-A^{Cnp1} or untagged CENP-A^{Cnp1}.

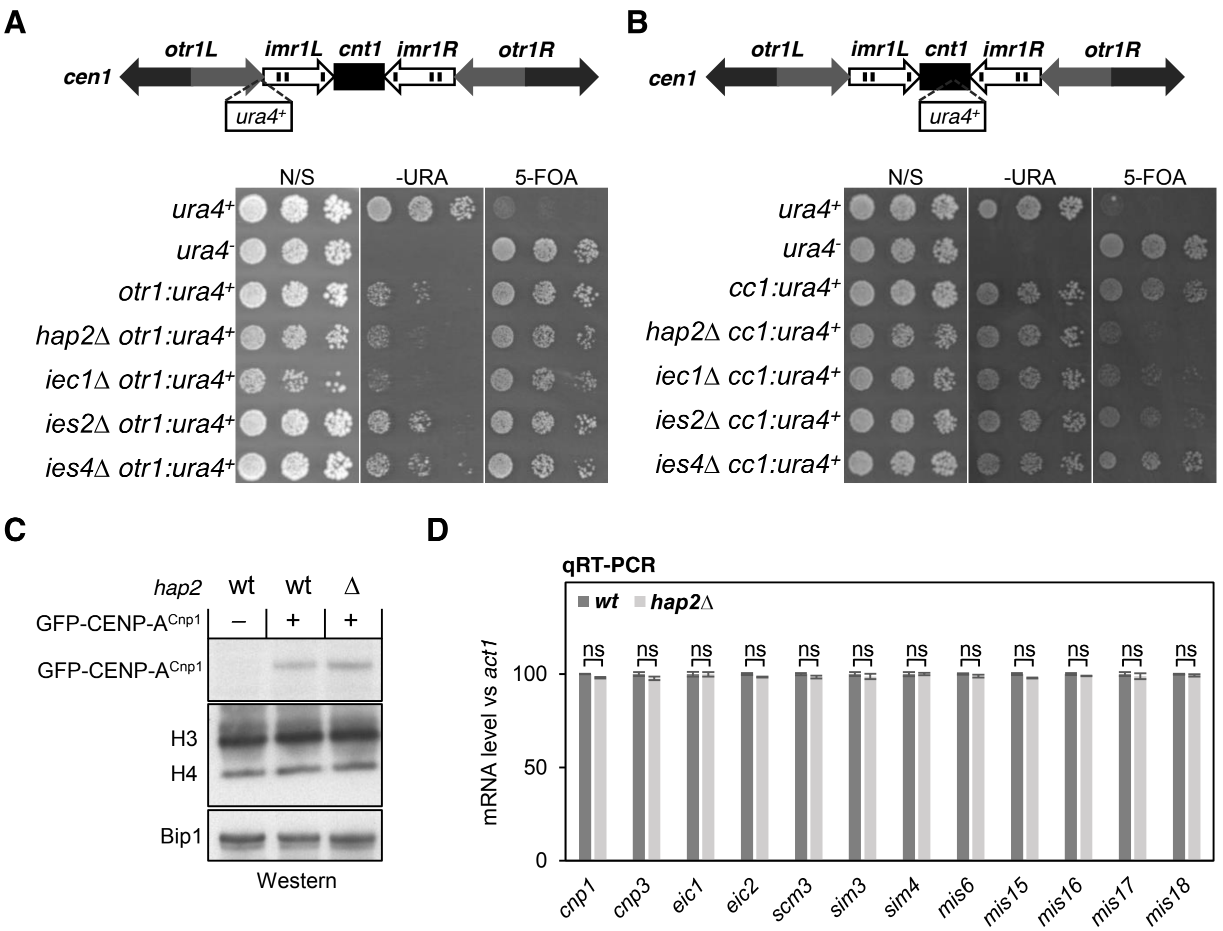

Figure S3. Loss of Hap2 does not affect cellular levels of CENP-A^{Cnp1} or mRNA levels of CENP-A^{Cnp1} chromatin-related genes. (A and B) Growth assay for at *cnt1-otr1:ura4^{+}* (A) or *cnt1-cc1:ura4^{+}* (B) in cells lacking the indicated Ino80 subunits. (C) Western analysis of GFP-CENP-A^{Cnp1}, histone H3, histone H4 and Bip1 (loading control) in whole cell lysates of wild-type and *hap2*Δ cells expressing GFP-CENP-A^{Cnp1} or untagged CENP-A^{Cnp1}. (D) qRT-PCR analysis to measure expression levels of the indicated genes which are known to affect CENP-A^{Cnp1} loading at fission yeast centromeres when mutated. Transcript levels are shown relative to the *act1^{+}* gene (n = 3). Significance of the differences observed between wild type and *hap2*Δ was evaluated using Student's t-test; * $p < 0.05$, ** $p < 0.005$; *** $p < 0.005$ and n.s., not significant.

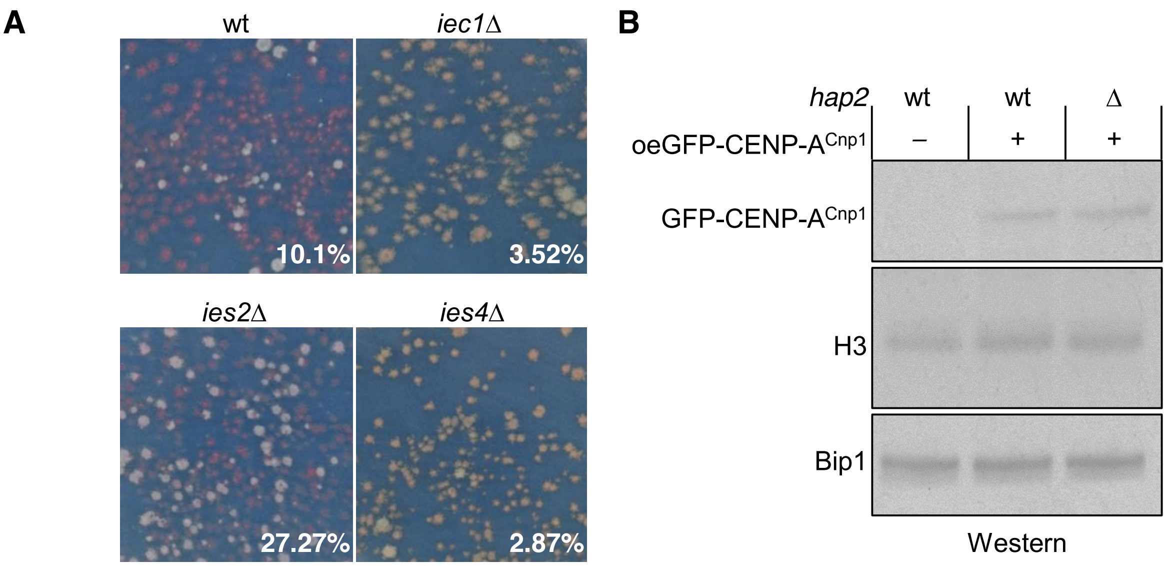

Figure S4. Cells lacking different Ino80 subunits show variable centromere establishment frequency. Overexpression of CENP-A^{Cnp1} is unaffected by the loss of Hap2. (A) *S. pombe* transformants containing pHcc2 minichromosome plasmids were replica-plated to low adenine non-selective plates: colonies retaining pHcc2 minichromosome plasmid have established centromeres are white/pale-pink, those that lose it are red. Representative plate showing established and non-established colonies and establishment frequency in wild type (n = 515), *iec1*Δ (n = 795), *ies2*Δ (n = 660) and *ies4*Δ (n = 802). (B) Western analysis of GFP-CENP-A^{Cnp1}, histone H3, histone H4 and Bip1 (loading control) in whole cell lysates of wild-type and *hap2*Δ cells over-expressing GFP-CENP-A^{Cnp1} or untagged CENP-A^{Cnp1}.

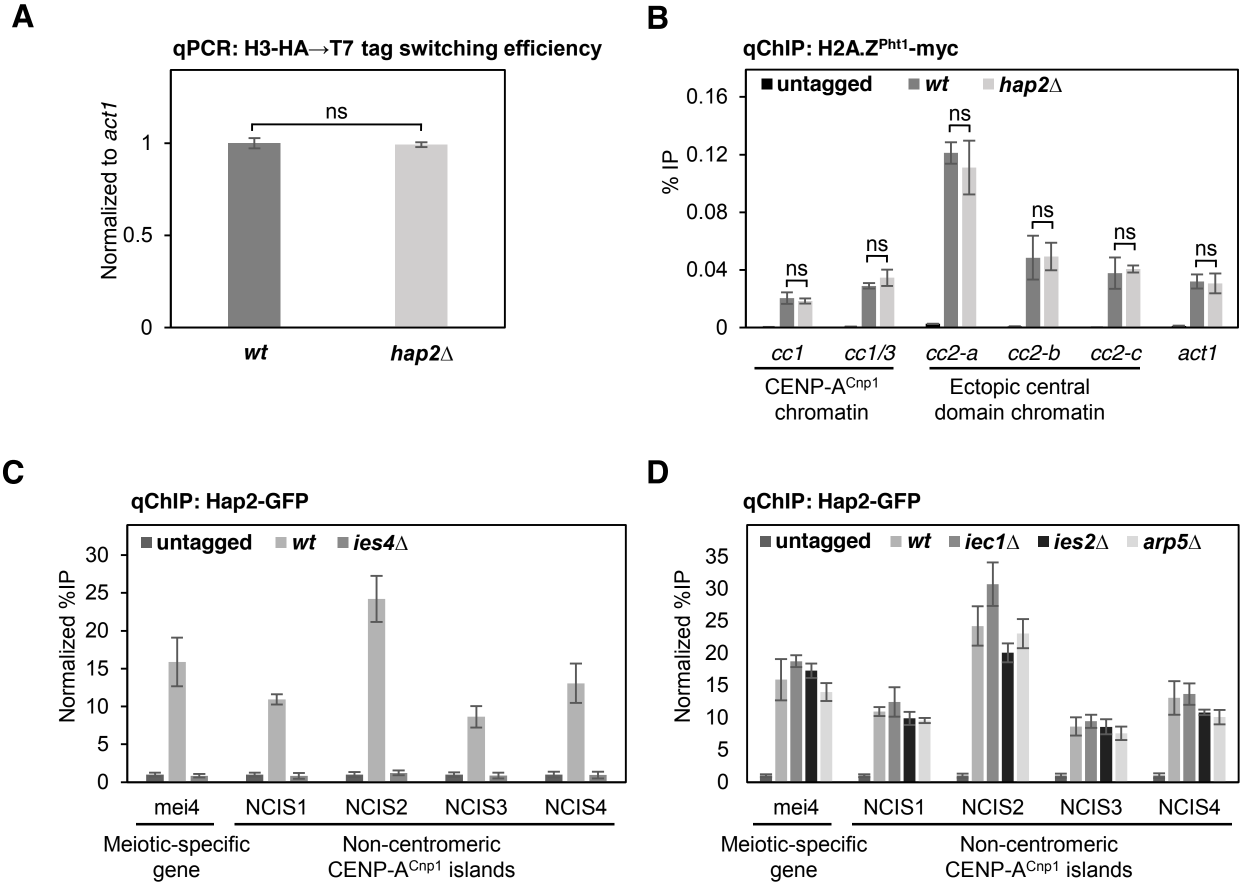

Figure S5. H3-HA➔T7 switching efficiency is not affected in cells lacking Hap2. H2A.Z^{Pht1} localization at central domain chromatin is independent of Hap2. Hap2-GFP association with non-centromeric CENP-A^{Cnp1} islands in Ies4-dependent. (A) qPCR for H3-HA→T7 switching efficiency of H3 tagged with HA and switched to T7 tag by β-estradiol induced Cre/loxP mediated recombination during *cdc25-22*/G2 block in 2 hours. Error bars indicate mean ± SD (n = 3). (B) qChIP for H2A.Z^{Pht1} at two locations within endogenous centromeres (*cc1* and cc1/3), three locations within ectopically inserted central domain 2 DNA (*cc2-a*, *cc2-b* and *cc2-c*) and non-centromere locus (*act1*$^{+}$), in wild type and *hap2*Δ. Error bars indicate mean ± SD (n = 3). Significance of the differences observed between wild type and *hap2*Δ was evaluated using Student's t-test in A; * $p < 0.05$, ** $p < 0.005$; *** $p < 0.005$ and n.s., not significant. (C and D) qChIP for Hap2-GFP at ectopic CENP-A^{Cnp1} islands (*NCIS1*, *2*, *3* and *4*) and meiotic-specific gene (*mei4*$^{+}$) in untagged, wild-type and *ies4*Δ (C) and in untagged, wild-type, *iec1*Δ*, ies2*Δ *and arp5*Δ (D) cells.

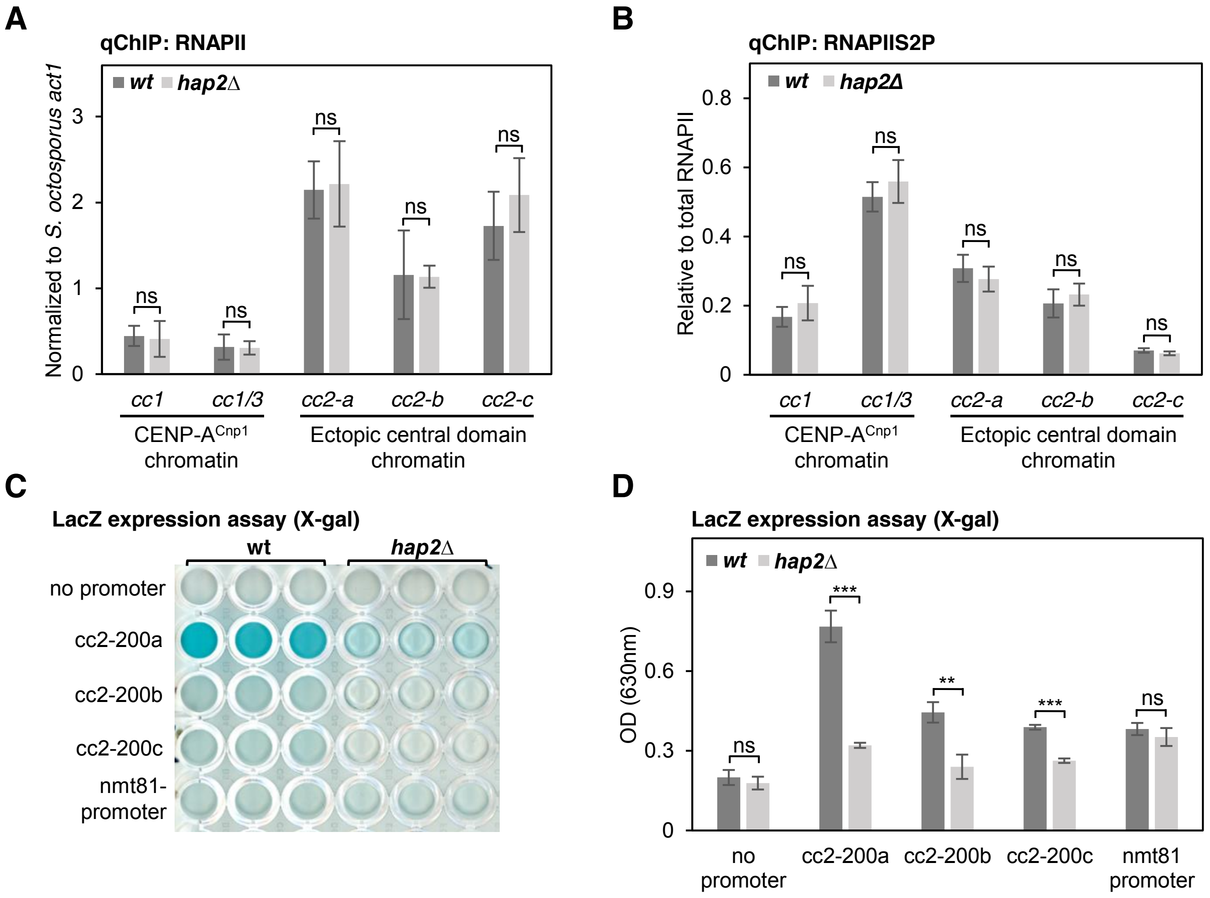

Figure S6. Transcription from centromeric DNA is reduced in the absence of Hap2. (A and B) qChIP for total RNAPII and RNAPIIS2P at two locations within endogenous centromeres (*cc1* and cc1/3) and three locations within ectopically-inserted central core 2 DNA (*cc2-a*, *cc2-b* and *cc2-c*) in wild type and *hap2*Δ in G3 (T140). Error bars indicate mean ± SD (n = 3). (C) Analysis of promoter activity from cc2 fragments (cc2-200a, cc2-200b and cc2-200c). The level of LacZ expression was assessed by measuring absorbance at 630 nm of cell lysates incubated with X-gal. nmt81: positive control with nmt81 promoter. (D) Quantification of levels of LacZ expression as assessed by measuring absorbance at 630 nm in samples shown in C (n = 3). Error bars indicate mean ± SD (n = 3). Significance of the differences observed between wild type and *hap2*Δ was evaluated using Student's t-test in A, B and D; * $p < 0.05$, ** $p < 0.005$; *** $p < 0.005$ and n.s., not significant.
